# Rescuing ESAT-6 Specific CD4 T Cells From Terminal Differentiation Is Critical for Long-Term Control of Murine Mtb Infection

**DOI:** 10.3389/fimmu.2020.585359

**Published:** 2020-11-06

**Authors:** Helena Strand Clemmensen, Niels Peter Hell Knudsen, Rolf Billeskov, Ida Rosenkrands, Gregers Jungersen, Claus Aagaard, Peter Andersen, Rasmus Mortensen

**Affiliations:** ^1^ Department of Infectious Disease Immunology, Statens Serum Institut, Copenhagen, Denmark; ^2^ Department of Health Technology, Technical University of Denmark, Lyngby, Denmark; ^3^ Department of Immunology and Microbiology, University of Copenhagen, Copenhagen, Denmark

**Keywords:** tuberculosis, vaccines, T cell differentiation, mouse models, ESAT-6, *Mycobacterium tuberculosis*, long-term protection, immunodominant antigens

## Abstract

In most cases, *Mycobacterium tuberculosis* (Mtb) causes life-long chronic infections, which poses unique challenges for the immune system. Most of the current tuberculosis (TB) subunit vaccines incorporate immunodominant antigens and at this point, it is poorly understood how the CD4 T cell subsets recognizing these antigens are affected during long-term infection. Very little is known about the requirements for sustainable vaccine protection against TB. To explore this, we screened 62 human-recognized Mtb antigens during chronic murine Mtb infection and identified the four most immunodominant antigens in this setting (MPT70, Rv3020c, and Rv3019c and ESAT-6). Combined into a subunit vaccine, this fusion protein induced robust protection both in a standard short-term model and in a long-term infection model where immunity from BCG waned. Importantly, replacement of ESAT-6 with another ESAT-6-family antigen, Rv1198, led to similar short-term protection but a complete loss of bacterial control during chronic infection. This observation was further underscored, as the ESAT-6 containing vaccine mediated sustainable protection in a model of post-exposure vaccination, where the ESAT-6-replacement vaccine did not. An individual comparison of the CD4 T cell responses during Mtb infection revealed that ESAT-6-specific T cells were more terminally differentiated than the other immunodominant antigens and immunization with the ESAT-6 containing vaccine led to substantially greater reduction in the overall T cell differentiation status. Our data therefore associates long-term bacterial control with the ability of a vaccine to rescue infection-driven CD4T cell differentiation and future TB antigen discovery programs should focus on identifying antigens with the highest accompanying T cell differentiation, like ESAT-6. This also highlights the importance of long-term readouts in both preclinical and clinical studies with TB vaccines.

## Introduction

Developing a vaccine preventing or mitigating pulmonary tuberculosis (TB) remains a high priority and simultaneously a significant scientific challenge. Despite mass vaccination with the current bacillus Calmette-Guérin (BCG) vaccine, BCG fails to control the current TB epidemic caused by infection with *Mycobacterium tuberculosis* (Mtb). With 1.5 million mortalities in 2018, TB is the leading cause of death from a single infectious pathogen (1). Encouragingly, two novel TB subunit vaccines have recently demonstrated signals of vaccine efficacy (VE); H4:IC31 VE 30.5% (2) and M72/ASO1_E_ VE 49.7% (3, 4). These results clearly demonstrate the potential of subunit vaccines and it is proposed that increased vaccine efficacy can be obtained with improved antigen composition and inclusion of additional Mtb antigens (5). However, efforts to design such vaccines are challenged by an incomplete knowledge of key antigens and mechanisms of protective immunity.

Most TB vaccines tested pre-clinically have been evaluated in relatively short models, where the animals are naïve to mycobacteria. Conversely, Mtb establish life-long chronic infections and a significant proportion of the population living in TB endemic regions have an already acquired Mtb infection thereby harboring a pre-existing memory response to Mtb (6, 7). A few of the more recent vaccine strategies have already been implemented to target individuals with active TB or latent tuberculosis infection (LTBI) (8) but it has proven more difficult to obtain vaccine-protection in animal models of post-exposure vaccination. The mechanisms behind protection in sensitized individuals is still debated (9). Given the established Mtb-primed immunity and presence of terminally differentiated CD4 T cells, the requirements for a vaccine to mediate protection in short preventive models do not necessarily mirror the demands for a vaccine to protect in lengthy chronic or post-exposure models. As future TB vaccines need to be suited for sustainable control of Mtb infection, the relevance of studying protective immunity against Mtb in long-term infection-models is essential.

Previous studies suggest that protective immunity against Mtb is associated with CD4 T cells’ ability to migrate into the lung parenchyma and suppress Mtb growth by being in direct contact with the infected cells (10, 11). CD4 T cell differentiation is a determinant of a T cells’ expression of pro-inflammatory cytokines and chemokine receptors and therefore also its capability to home to infected tissues. Importantly, less differentiated KLRG1^-^ CD4 T cells producing IL-2 are shown to have a higher proliferative capacity, which is associated with long-term control of Mtb infection (12–14). In contrast, terminally differentiated CD4 T cells expressing KLRG1 are short-lived, accumulate in the vasculature (14, 15), migrate poorly into the lung and therefore fail to suppress Mtb growth (15–20). Induction or maintenance of less differentiated CD4 T cells might therefore be an important component of long-term vaccine immunity.

Only few antigens have been selected for clinical vaccine candidates and little is known about how long-term chronic infection might affect antigen-specific CD4 T cell populations. Early secretory antigenic target-6 (ESAT-6) is a well-known immunodominant antigen that is highly recognized in humans with latent and active TB (21–23). It is used in the majority of immunological studies, incorporated into several vaccine designs (24–28) and has also been suggested to be required for post-exposure protection of the H56 subunit vaccine (9, 26, 29). Recently, it was demonstrated that natural Mtb infection drives ESAT-6 specific CD4 T cells towards a state of terminally differentiation in contrast to another well-known antigen, Ag85B (30). Given that the vaccine candidates under clinical and pre-clinical investigation incorporate different immunodominant antigens (1), the aims of the current study were therefore 1) to explore to what extent immunodominant antigens, similar to ESAT-6, confer protection in models of long-term chronic infection or post-exposure vaccination, and 2) to investigate whether the state of CD4 T cell differentiation influences long-term protection of vaccine antigens.

Herein, we identified four of the most immunodominant Mtb antigens measured by IFN-γ responses and tested their combined ability to provide sustainable protection in murine models of chronic Mtb infection. Importantly, we found that only one of these antigens, ESAT-6, was essential for long-term protection despite displaying equal protective efficacy in the standard short-term model. Our data suggest that this is linked to differential antigen-specific CD4 T cell differentiation driven by the infection. Therefore, vaccination with antigens of highest T cell differentiation, exemplified by ESAT-6, has the greatest potential for rescuing terminal differentiation and inducing long-term protection. As many TB vaccines incorporate immunodominant antigens, these new insights should be explored in human studies and guide future vaccine design.

## Materials and Methods

### Mice

Six to eight week old female CB6F1 mice (BALB/c x C57BL/6) were purchased from Envigo, The Netherlands. Mice were randomly assigned to cages of eight at the day of arrival. Before initiating any experimental procedure, mice had at least one week of acclimation. During the experiment mice were fed with irradiated Teklad Global 16% Protein Rodent Diet (Envigo, 2916C) and water ad libitum.

Mice were housed in Biosafety Level (BSL) II in individual ventilated cages (Scanbur, Denmark) and had access to nesting material (enviro-dri and soft paper wool; Brogaarden) as well as enrichment (aspen bricks, paper house, corn, seeds and nuts; Brogaarden). At the day of challenge, cages with mice were transferred to BSL-III where they were housed until termination of the experiment. All experimental protocols were initially reviewed and approved by a local ethical committee at Statens Serum Institut (SSI). Experimental procedures were conducted in accordance with the regulations set forward by the Danish Ministry of Justice and Animal Protection Committees under license permit no. 2014-15-2934-01065 and in compliance with the European Union Directive 2010/63/EU.

### Immunizations and Recombinant Proteins

CB6F1 mice were vaccinated three times subcutaneously (s.c.) at the base of the tail with two weeks interval. Dose and time for vaccinations depended on the experimental model, immunizations were given at week 0, 2, and 4 (preventive model) and at week 14, 16, and 18 after the 1^st^ Mtb challenge (re-infection model). Vaccination with 2x10^5^ CFU BCG Danish were given once at week 0.

The following recombinant fusion proteins were expressed and purified: H83 (MPT70-ESAT-6-Rv3020c-Rv3019c) and H89 (MPT70-Rv1198-Rv3020c-Rv3019c), as well as individual antigens. The DNA constructs were codon-optimized for expression in *E. coli* before insertion into the pJ 411 expression vector (ATUM, Menlo Park, CA, US). In all proteins, we added a His-tag at the N-terminal end (MHHHHHH-). After transformation into *E. coli* BL21 (DE3) (Agilent, DK), protein expression was induced with 1 mM isopropyl ß-d-1-thiogalactopyranoside in 3-liter cultures, and the proteins were purified from inclusion bodies by metal chelate chromatography followed by anion-exchange chromatography. The purity was assessed by SDS-PAGE followed by Coomassie staining and was above 95%.

Mice were immunized with either recombinant fusion proteins; H83 or H89 or individual antigens (ESAT-6, MPT70, Rv3020c*, Rv3019c, or Rv1198*). *In single-antigen immunizations mice were vaccinated with the Rv1198 homologue Rv3619c (**Figures 3A, B**) sharing 98% amino acid identity, including full conservation of the dominant murine epitope (31, 32) and Rv0287 instead of Rv3020c (**Figures 2A, B**) that share 92% sequence identity and elicits known cross-reactivity after vaccination (33–35). Recombinant proteins were diluted in Tris-HCL buffer (pH 7.2) and adjuvanted with cationic adjuvant formulation 1 (CAF01) which consists of dimethyldioctadecylammonium (DDA) and trehalose dibehenate (TDB) in a ratio 250 µg DDA per/50 µg TDB (36). As control, mice were either left non-vaccinated, vaccinated with Tris-HCL buffer (saline) or vaccinated with adjuvant only (CAF01).

### Experimental TB Mouse Models

In the present study we present data from two experimental TB mouse models.

**The preventive model:** Mice were left non-vaccinated or vaccinated s.c. three times with CAF01 or 0.5–2.0 µg H83 or H89 and 10.0 µg MPT70, ESAT-6, Rv3020c, Rv3019c, or Rv1198 at week 0, 2, 4, and challenged with Mtb 6 weeks after the 3^rd^ vaccination (week 10). Colony forming units (CFU) were determined at different time points.

**The re-infection model:** Mice were infected by the aerosol route with 25-50 CFU Mtb Erdman (1^st^ infection, week 0). Four weeks into the infection, mice were administered 0.1 g L^−1^ rifabutin (RIF) and 0.1 g L^−1^ isoniazid (INH) (BD) in their drinking water for 12 weeks (from week 4 to week 16) to clear the primary infection. At week 14, 16, and 18, mice were immunized with 0.5 µg H83 or H89 three times s.c. or left non-vaccinated. Mice were allowed to rest 8 weeks after end of antibiotic treatment (RIF and INH) and then challenged with 50–100 CFU Mtb Erdman (2^nd^ infection, week 24) and protective efficacy was assessed 4 and 8 weeks into 2^nd^ Mtb infection.

### Mtb Infections and CFU Enumeration

Mtb Erdman (ATCC 35801/TMC107) were cultured in Difco ™ Middlebrook 7H9 (BD) supplemented with 10% BBI ™ Middlebrook ADC Enrichment (BD) for two-three weeks using an orbital shaker (~110 rpm). Bacteria were harvested in log phase and stored at -80 degrees until use. Before used in experiment the concentration of the bacterial stock was determined by plating in triplicate. For aerosol infections, the vial of Mtb was thawed, sonicated for five minutes, thoroughly suspended with a 27G needle to remove clumps and mixed in PBS to the desired concentration.

In the preventive model, mice were rested 6 weeks after the third immunization prior to challenge. Mice were challenged with 0.2–0.5 x 10^6 CFU/ml (around 50–100 CFUs) Mtb Erdman by the aerosol route using a Biaera exposure system controlled *via* AeroMP software. Each aerosol round could take up to 80 mice.

To enumerate bacteria in the lungs of mice after infection, left lobes from individual mice was homogenized with GentleMACS M-tubes (Miltenyi Biotec) in 3 mL MilliQ water containing PANTA™ Antibiotic Mixture (BD, cat.no. #245114). The homogenate was serially diluted, plated and grown on 7H11 plates (BD) for approximately 14 days at 37 degrees and 5% CO_2_. CFU were counted, log-transformed to normalize data and shown as log_10_ CFU per whole lung. Whenever possible a cutoff of 10 colonies were set to minimize variability and errors due to plating.

### *In vivo* CD45 Staining of Intravascular Leucocytes

Prior to euthanization, 2.5 µg fluorescein isothiocyanate (FITC) labelled CD45 antibody (BD Pharmingen, clone 104; 553772) was diluted in 250 µl PBS and injected intravenously in the tail vein of mice. Three minutes after injection, mice were euthanized by cervical dislocation and lungs were harvested for further processing as described below.

### Preparation of Single Cell Suspensions

Spleens or lung were aseptically harvested from euthanized mice. Lungs were first homogenized in Gentle MACS tubes C (Miltenyi Biotec) followed by 1 h of collagenase-digestion (Sigma Aldrich; C5138) at 37 degrees, 5% CO_2_. The lung homogenate and spleens were forced through 100 µm cell strainers (BD Biosciences) with the stopper from a 5 ml syringe (BD) and washed twice with cold RPMI medium (Gibco; RPMI-1640) by centrifuging 5 min at 1,800 rpm. Cells were finally re-suspended in enriched RPMI medium [RPMI-1640, 10% heat-inactivated FCS (Biochrom Gmbh), 10 mM Hepes (Invitrogen), 2 mM L-Glutamine (Invitrogen), 1 mM Natriumpyruvate (Invitrogen), 1× Non-essential amino acids (MP Biomedicals, LLC), 5×10^-5^ M 2-mercaptoethanol (Sigma-Aldrich), and PenStrep (Gibco)]. Cells were counted using an automatic Nucleocounter™ (Chemotec) and adjusted to 2x10^5^ cells/well for ELISA and 1-2x10^6^ cells/well for flow cytometry.

### *In vitro* Re-Stimulation and Intracellular Cytokine Staining

For intracellularly cytokine staining (ICS), cells were restimulated with 2 µg/ml antigen or medium in the presence of 1 μg/ml anti-CD28 (clone 37.51) and anti-CD49d (clone 9C10-MFR4.B) in 96V-bottom TCT microtiter plates (Corning; 3894) for 1 hour at 37 degrees and 5% CO_2_. This was followed by the addition of 10 µg/ml Brefeldin A (Sigma Aldrich; B7651-5mg) and another 5–6 h incubation at 37 degrees, 5% CO_2_ after which cells were kept at 4 degrees until staining. For Rv3020c we restimulated with the orthologue subfamily member Rv0287 (EsxG) (**Figures 2D**, **4A, B, D**).

Prior to staining, cells were washed with FACS buffer (PBS+1% FCS) and subsequently stained with fixable viability dye eflour780 (eBioscience;65-0865-14;clone:n/a) and surface markers diluted in brilliant stain buffer (BD Horizon; 566349) using anti-CD3 Bv650 (Biolegend;100229;clone:17A2), anti-CD4 Bv510 (Biolegend;100553;clone:RM4-5), anti-CD44 Bv786 (BD Bioscience; 563736;clone:IM7), anti-KLRG1 Bv711 (Biolegend;138427;clone:2F1), and anti-PD-1 Bv421 (Biolegend;135217; clone:29F.1A12). Fixation and permeabilization was carried using the Fixation/Permeabilization Solution Kit (BD Cytofix/Cytoperm; 554714) as per manufacturer’s instructions followed by ICS with anti-IFN-γ PE-Cy7 (BD Biosciences;557649;clone:XMG1.2), anti-IL-2 APC (eBioscience;17-7021-82;clone:JES6-5H4) and anti-TNFα PE (BD Biosciences;554419;clone:MP6-XT22). Fluorescence minus one controls were performed for CD3, CD44, KLRG1 and PD-1 on pooled cells to set surface marker gates. Restimulation with ionomycin in conjunction with phorbol myristate acetate was included as positive control. Cells from the murine studies were analyzed using a BD LRSFortessa and the FSC files were afterwards manually gated with FlowJo v10 (Tree Star). The immune responses shown for the lung corresponds to the whole organ and we first distinguish between the CD45^+^ and CD45^-^ cells after gating for antigen-specific CD4 T cells (**Supplementary Figure 1**).

### Epitope Binding Predictions

A pre-selection of Mtb antigens was performed based on two previous genome-wide epitope screening studies in human LTBI donors (33, 37). From the initial screening many of the antigens were homologues proteins meaning that identical peptides could be found in several antigens. In this case, only one of the homologues proteins were selected for the murine screening. Gene expression data was used to determine which of the two-three homologues proteins that were highest expressed by Mtb (38). A total of 62 prevalently recognized Mtb antigens were selected and in silico predicted for murine CD4 T cell epitopes using the NetMHCII 2.3 Server (39). The server predicted H2-IA strong-binding epitopes by creating a peptide library of 1x10^6^ random natural peptides and ranking the peptides according to binding affinity. Strong binding epitopes were defined as having a binding affinity threshold below 2% of the total set of peptides. The computational screening resulted in 318 potential epitopes, with a typical core of 8-9 amino acids. To maximize the chance of covering the true epitopes, we synthesized 17-mer peptides (JPT, Germany) spanning the core plus an additional 4-5 amino acids at each end (**Supplementary Table 1**).

### IFN-γ Sandwich ELISA

First, splenocytes was adjusted to a cell concentration of 2x10^5^ cells/ml and restimulated together with recombinant protein or peptides in a round bottom plate for 3 days. A sandwich ELISA was performed on the culture supernatants to determine the concentration of total IFN-γ. Microtiter plates (96-well; Maxisorb; Nunc) were coated with 1 µg/ml monoclonal rat anti-murine IFN-γ (clone R4-6A2; BD Pharmingen) diluted in carbonate buffer. Free binding sites were blocked with 2% (w/v) skimmed milk powder in PBS. Culture supernatants were harvested from lymphocyte cultures after 72 h of incubation at 37 degrees, 5% CO_2_. Microtiter plates were incubated with diluted samples overnight whereafter IFN-γ was detected with a 0.1 µg/ml biotinylated rat anti-murine Ab (clone XMG1.2; BD Pharmingen) and 0.35 µg/ml HRP-conjugated streptavidin (Invitrogen Life Technologies). The enzyme reaction was developed with 3,3’,5,5’-tetramethylbenzidine, hydrogen peroxide (TMB Plus; Kementec) and stopped with 0.2 M H_2_SO_4_ solution. Recombinant IFN-γ (BD Pharmingen) was used as standard. Plates were read at 450 nm with 620 nm background correction using an ELISA reader (Tecan Sunrise).

### Statistical Analyses

All graphical visualizations and statistical tests were done using GraphPad Prism v8. The significance of difference between two antigen restimulations from the same group was assessed with a two-tailed paired t-test (**Figures 2A**, **3A**). When more than two vaccine groups were compared relative to the saline group, we evaluated the significance of difference by One-Way Analysis of Variance (ANOVA) using Dunnett’s multiple comparison test (**Figure 2B**). To test for significant differences across all groups, we performed a One-Way ANOVA with Tukey’s multiple comparison test (**Figures 2E**, **3B**). In cases where we compared more than two antigen restimulations within the same group, we performed a Brown Forsythe and Welch ANOVA test with Dunnett’s multiple comparison test which did not assume equal standard deviations (**Figures 2D**, **3D**). Finally, significant differences in vaccine protection between multiple vaccine groups and different time points for CFU readouts, were evaluated by a Two-Way ANOVA with Tukey’s multiple comparison test (**Figures 3F, 3H**). The type of statistical test performed, degree of freedom (df), F-value (DFn, DFd), W-value (DFn, DFd) or t-value together with the exact p-value are indicated in the individual figures and figure legends. A p-value below 0.05 was considered a significant difference.

## Results

### Identification of Immunodominant Mtb Antigens During Chronic Infection

ESAT-6 is an immunodominant antigen with known vaccine potential (2, 24–28, 40). Based on previous reports (29, 30) we hypothesized that ESAT-6 plays a key role in late-stage vaccine protection and therefore sought to identify other immunodominant antigens for comparison both as stand-alone antigens as well as in larger fusion proteins.

**Figure 1:**
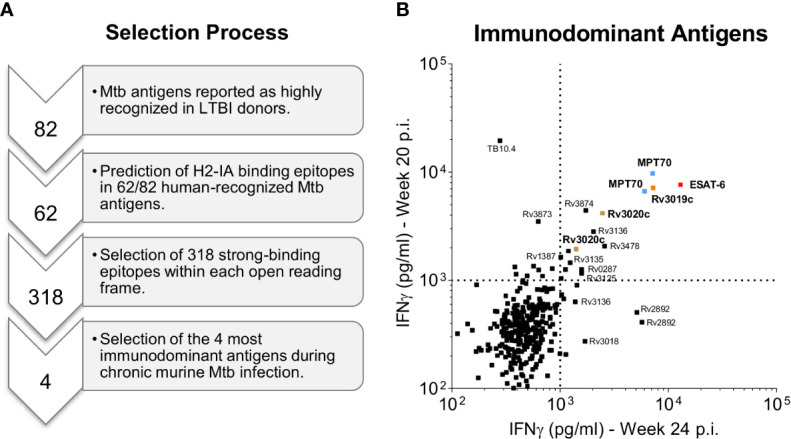
Selection of immunodominant antigens during chronic Mtb infection. **(A)** A pre-selection of Mtb antigens were performed on the basis of two previous genome-wide epitope screening studies in human LTBI donors (33, 37). A total of 62 Mtb antigens were selected and *in silico* predicted for the presence of murine CD4 T cell epitopes using the NetMHCII 2.3 Server (39). The computational screening resulted in 318 potential murine CD4 T cell epitopes that were synthesized as 17-mer peptides (**Supplementary Table 1**). Finally, the four most immunodominant antigens were selected based on high CD4 T cell recognition of the synthesized Mtb peptides by CB6F1 mice during late chronic Mtb infection (20 and 24 weeks post challenge) as presented in b. **(B)** In two independent experiments splenocyte culture supernatants were harvested after 3 days of culture with individual peptides and the level of IFN-γ was measured. The top four immunodominant antigens (1^st^ quadrant) are highlighted in bold and color coded: MPT70 (Rv2875): blue, ESAT-6 (Rv3875): red, Rv3020c: brown and Rv3019c: orange. Each squared dot represent a single peptide response.

Our first criterion for antigen selection was strong immune recognition in humans as well as mice carrying a long-term chronic Mtb infection. We first performed a pre-selection of antigens based on gene expression data (38) as well as human recognition in two previous studies with human donors (33, 37). In total we selected 62 prevalently recognized Mtb antigens (see materials and methods), from which 318 murine H2-IA-binding epitopes were *in silico* predicted (**Figure 1A**). We next infected CB6F1 mice with Mtb Erdman by the aerosol route and used predicted peptide epitopes for restimulating splenocytes during late chronic infection (≥20 weeks post infection). To ensure reproducible results, we performed two independent experiments (**Supplementary Table 1**) and selected the top 4 antigens with highest responding peptides in both studies for efficacy testing; MPT70 (Rv2875), ESAT-6 (Rv3875), EsxS (Rv3020c) and EsxR (Rv3019c) (**Figure 1B**). Noteworthy, Rv3125c (PPE49), Rv3135 (PPE50), Rv3136 (PPE51), Rv3478 (PPE60), Rv3873 (PPE68), Rv3874 (CFP-10) were among other antigens found to be immunodominant during chronic TB in CB6F1 mice.

**Figure 2:**
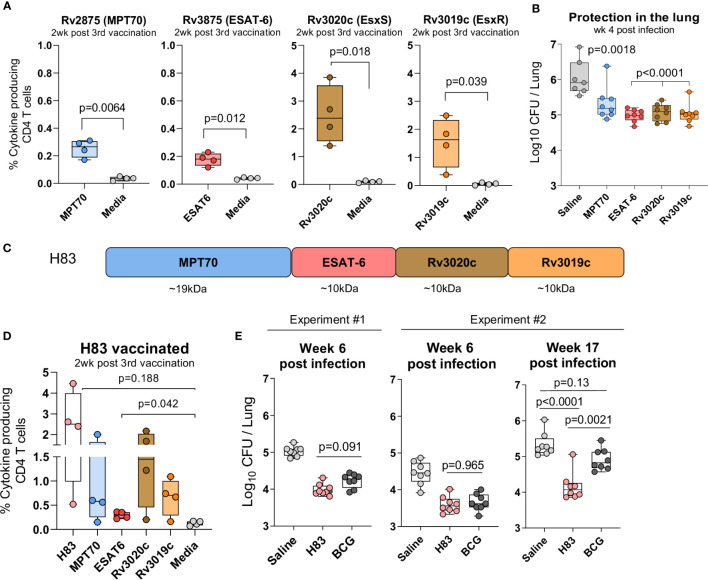
H83, a fusion protein of immunodominant antigens, confer robust long-term protection. Female CB6F1 mice were vaccinated with 10 µg of either MPT70, ESAT-6, Rv3020c, Rv3019c, or Tris-HCL buffer three times s.c. at the base of the tail. **(A)** Immunogenicity of single antigens were assessed two weeks after 3^rd^ immunization by stimulating splenocytes *in vitro* with individual antigens or media. Figures represent percentage of cytokine producing (TNFα and/or IFN-y and/or IL-2) CD4 T cells after intracellular cytokine staining (ICS) (n = 4). The gating strategy for antigen-specific CD4 T cells is shown in **Supplementary Figure 1**. Paired t-test, two-tailed, t-values (6.84, 5.52, 4.71, 3.51, df = 3). **(B)** Vaccine-mediated protection were determined by counting the bacterial numbers in the lungs of MPT70-, ESAT-6-, Rv3020c-, and Rv3019c-vaccinated mice at week 4 post Mtb Erdman infection (n = 7–8). Colony forming units (CFUs) for one saline mouse was too numerus to count. One-way ANOVA with Tukey’s multiple comparison test, F-value (5, 41) = 41.59. **(C)** The fusion protein, hybrid 83 (H83), was composed of MPT70, ESAT-6, Rv3020c, and Rv3019c in the mentioned order. **(D)** Percentage of cytokine-producing CD4 T cells after H83 vaccination measured by ICS. Splenocytes were restimulated with H83, MPT70, ESAT-6, Rv3020c, Rv3019c, or media two weeks after 3^rd^ immunization (n = 4). Brown-Forsythe and Welch ANOVA tests with Dunnett’s multiple comparison test, F-value (5.000, 6.606) = 4.351, W-value (5.000, 7.664) =5.975. **(E)** CB6F1 mice were vaccinated with 2 µg H83 three times or once with 2x10^5^ CFU BCG Danish. Six weeks after 3^rd^ subunit vaccination or ten weeks after BCG immunization, mice were challenged with Mtb. Bacterial numbers were determined in the lungs of H83- and BCG-vaccinated mice in two individual experiments at week 6 and one experiment at week 17 post Mtb infection (n = 8). One-way ANOVA with Tukey’s multiple comparison test was performed, and the exact p-values indicated, F-values (3, 28) = 44.02, 23.07, 21.26. Data shows box plots with whiskers indicating minimum and maximum values.

The second criterion for the immunodominant antigens, was immunogenicity and protective efficacy as stand-alone vaccines in the standard preventive mouse model. Groups of CB6F1 mice were immunized three times s.c. with either MPT70, ESAT-6, Rv3020c, or Rv3019c formulated in the CAF01 adjuvant (36, 41). Two weeks after the 3^rd^ immunization, the frequency of cytokine-producing (TNFα, IFN-γ, or IL-2) CD4 T cells recognizing its respective vaccine antigen was determined by flow cytometry and all four antigens induced significant immune responses (**Figure 2A**). Notably, despite being immunodominant during infection, both MPT70 and ESAT-6 were less immunogenic in the context of single protein vaccination compared to Rv3019c and Rv3020c. Thus, Rv3020c and Rv3019c induced substantially higher immune responses after vaccination (2.503 ± 0.523 and 1.535 ± 0.443) compared to MPT70 and ESAT-6 (0.235 ± 0.031 and 0.178 ± 0.023) even though all the antigens were similar in size (10–20 kDa) and had similar magnitudes of response after Mtb infection (**Figure 2A**). According to the standard short-term preventive model, vaccinated mice received an aerosol challenge with Mtb Erdman, and bacteria were enumerated in the lungs four weeks later (peak infection). Notably, all the selected antigens induced significant protection in this model, MPT70 (-Δlog 0.71), ESAT-6 (-Δlog 1.06), Rv3020c (-Δlog 0.97), and Rv3019c (-Δlog 1.00) (**Figure 2B**).

In summary, we selected 318 predicted mouse Mtb-derived epitopes from human recognized antigens and tested their reactivity in mice with a chronic Mtb infection. We identified three antigens, MPT70, Rv3019c, and Rv3020c that were equally or more immunogenic than ESAT-6 and provided similar short-term protective efficacy after single-antigen immunization.

### A Fusion Protein of the Immunodominant Antigens Confer Robust Long-Term Protection

We next investigated whether this combination of immunodominant antigens would provide long-term protection when formulated as a single fusion protein vaccine, H83 (**Figure 2C**). As previously, mice were vaccinated three times with H83 in CAF01 and the magnitude of the antigen responses were analyzed two weeks after vaccination. Here, we observed similar magnitudes of immune responses compared to single protein immunization, indicating that the immune hierarchy is dependent on antigen-intrinsic factors rather than the order of antigens in the fusion molecule (**Figure 2D**). We then assessed protective efficacy in a standard preventive experiment with readout six weeks after infection and observed that H83 and BCG induced similar levels of protection at this time point, -Δlog 0.86 and 0.80, respectively (experiment #1, **Figure 2E**). After this, we performed a second experiment that included a late time point during chronic infection, where CD4 T cells become terminally differentiated (12, 15, 42). Data from this experiment confirmed that H83 and BCG induced similar levels of protection at the early time point (-Δlog 1.04 vs. 0.80), but as the infection progressed into the late chronic phase (week 17), immunity from BCG waned (-Δlog 0.42) whereas H83 immunized animals remained significantly protected (-Δlog 1.16). This demonstrates first, that adjuvanted protein subunit vaccines might have an advantage over BCG in long-term protection, despite providing similar early protection and secondly, that the combination of immunodominant antigens selected for this study conferred sustained bacterial control.

### ESAT-6 Is Critical for Long-Term Vaccine Protection

ESAT-6 is a known antigen with well-described protective capacity (29, 43), and it has been suggested to have a unique potential in post-exposure vaccination (29). However, it is unknown whether antigens have differential protective capacity against long-term chronic infections and whether ESAT-6 might play a key role in this setting compared to other immunodominant antigens. We therefore asked if the long-term protection offered by H83 was dependent on ESAT-6 and approached this question by investigating whether ESAT-6 could be replaced by a similar ESX-related antigen.

**Figure 3:**
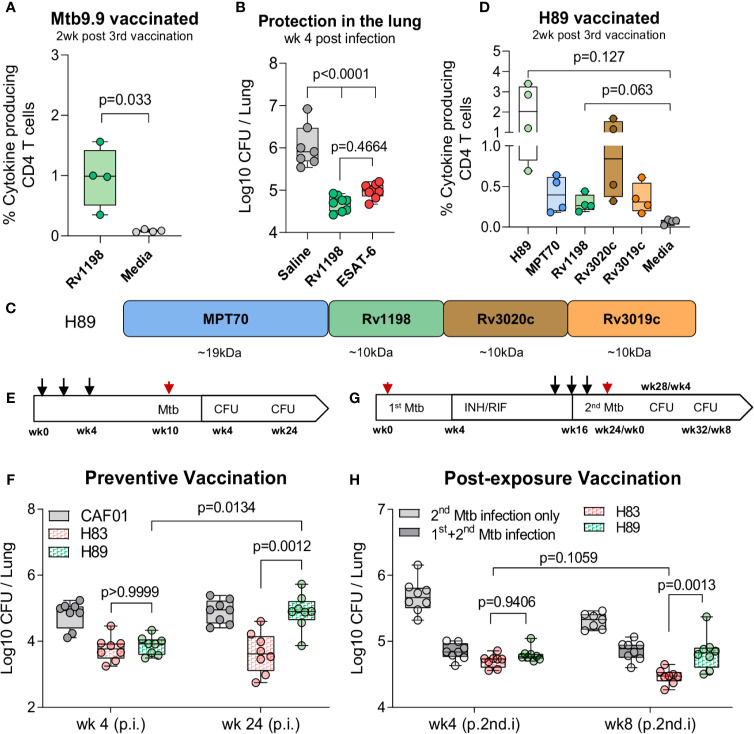
H83, but not H89, mediate long-term protection in chronically infected mice. **(A)** Percentage of of Rv1198-specific CD4 T cells was assessed in Mtb9.9-vaccinated CB6F1 mice by ICS. Splenocytes were restimulated Rv1198 pepmix or media two weeks post 3^rd^ vaccination (n = 4). Paired t-test, two-tailed, t-values (3.75, df = 3). **(B)** The bacterial burden were determined in the lungs of saline, Rv1198 and ESAT-6-vaccinated mice 4 weeks post Mtb infection (n = 8) as shown by CFUs relative to mice immunized with saline. An Ordinary One-way ANOVA with Tukey’s multiple comparison test, F-value (5, 41) = 41.59, was performed and the exact p-values indicated. **(C)** H89 was constructed by replacing ESAT-6 with an ESAT-6 like antigen, Rv1198, in a vaccine consisting of the same highly immunogenic antigens as in H83. **(D)** Percentage of antigen-specific CD4 T cells in H89-vaccinated mice. Splenocytes were restimulated with H89, MPT70, Rv1198, Rv3020c, Rv3019c, or media two weeks after 3^rd^ vaccination (n = 4). Brown-Forsythe and Welch ANOVA tests with Dunnett’s multiple comparison test, F-value (3.000, 3.045) = 8.660, W-value (3.000, 5.882) = 7.711. **(E)** Schematic overview of the standard preventive model. Mice were vaccinated s.c. three times with the CAF01 adjuvant only or 0.5 µg H83 or H89 at the base of the tail with two-week interval (black arrows) and challenged with 50–100 CFU Mtb Erdman 6 weeks after the 3^rd^ vaccination (red arrow). CFUs were determined 4 and 24 weeks after Mtb challenge. **(F)** Early (week 4) and late chronic (week 24) vaccine protection of H83 and H89 in a preventive setting (n = 8). A Two-Way ANOVA with Tukey’s multiple comparison test comparing cell means regardless of rows and columns, F_interaction_ (3, 56) = 3.134, F_row_ (1, 56) = 6.819, F_column_ (3, 56) = 12.77. Post infection (p.i.) **(G)** Schematic overview of the re-infection model (post-exposure vaccination). CB6F1 mice were infected by the aerosol route with 25-50 CFU Mtb Erdman (1^st^ infection, red arrow). One group of mice were left uninfected. Four weeks into the infection mice were administered antibiotics in their drinking water for 12 weeks to clear the primary infection. At week 14, 16, and 18, mice were immunized with 0.5 µg H83 or H89 s.c. at the base of the tail or left non-vaccinated (black arrows). All mice were then challenged with 50–100 CFU Mtb Erdman (2^nd^ infection, red arrow) 6 weeks post 3^rd^ vaccination and level of lung infection was quantified 4 and 8 weeks into 2^nd^ Mtb infection. **(H)** In the reinfection model: Total lung CFU in non-vaccinated mice (2^nd^ infection only), Mtb memory mice (1^st^ & 2^nd^ infection), H83 and H89-vaccinated mice 4 and 8 weeks after the 2^nd^ Mtb infection (n = 8). Post 2^nd^ infection = (p.2^nd^.i). A Two-Way ANOVA with Tukey’s multiple comparison test comparing cell means regardless of rows and columns, F_interaction_ (3, 55) = 5.503, F_row_ (1, 55) = 11.48, F_column_ (3, 55) = 85.10. Exact p-values are indicated. Data shows box plots with whiskers indicating minimum and maximum values.

Our criteria for a suitable ESAT-6-replacement were: 1) Belong to the ESX family proteins, 2) Reported as immunodominant protein, and 3) Induce robust protection on single-antigen level in the standard short-term model. As the vaccine already represented antigens from the ESX-1 and ESX-3 secretion systems, we selected the ESAT-like protein 4, also known as Rv1198 (EsxL), which belongs to the ESAT-6 subfamily Mtb9.9 encoded by the ESX-5 secretion system (34, 35). Together with the closely related EsxV (Rv3619c), Rv1198 has previously been described as an immunodominant antigen in LTBI individuals (33, 37) and shown protective efficacy as a component of the ID93/GLA-SE vaccine (44, 45). Like ESAT-6, Rv1198 is also a low molecular weight protein, which is expressed on the level with ESAT-6 *in vitro* (46) and among the top 15% highest expressed genes *in vivo* (21). We first investigated if Rv1198 met the criteria for immunogenicity and protective efficacy in our model system. In the spleens of Rv1198-vaccinated mice, we found a frequency of 0.973 ± 0.247 antigen-specific CD4 T cells after vaccination (**Figure 3A**), which was higher than the observed vaccine responses for ESAT-6 (**Figure 2A**). Six weeks after vaccination, mice were challenged with Mtb Erdman and the bacterial burden at week 4 was significantly reduced in Rv1198-vaccinated mice (-Δlog 1.36) and comparable to the level for ESAT-6 (-Δlog 1.06) (**Figure 3B**), supporting that ESAT-6 and Rv1198 have similar vaccine properties in terms of both immunogenicity and short-term protective capacity.

To investigate the effect of exchanging ESAT-6 with Rv1198 on long-term immunity, we constructed a fusion protein, H89, which held Rv1198 in the same position as ESAT-6 in H83 (**Figure 3C**). When mice were vaccinated with H89, we detected a comparable magnitude of Rv1198-specific CD4 T cells to ESAT-6-specific CD4 T cells in H83-vaccinated mice (**Figure 2D**) indicating that the proteins were equally immunogenic within the subunit vaccines (**Figure 3D**). Vaccinated mice where challenged with Mtb Erdman and the bacterial burdens were assessed in the lungs of mice 4 and 24 weeks post challenge (**Figure 3E**). As expected, both H83 and H89 induced robust and significant protection over saline (-Δlog 1.03 and -Δlog 0.93) at the early time point. However, at week 24 the bacterial load in H89-vacccinated mice had reached the levels of the saline group (-Δlog 0.01), whereas H83-vaccinated mice remained significantly protected (-Δlog 1.25), indicating that ESAT-6 was required for sustainable protection (**Figure 3F**). Epitope mapping of H83 and H89 vaccinated mice showed that the vaccines induced highly similar epitope patterns supporting that the differences in protective efficacy likely was due to inclusion of ESAT-6 and not differences in proteolysis and/or antigen presentation (**Supplementary Figure 2**).

We finally investigated whether ESAT-6 was also required for vaccine-mediated protection in animals with pre-existing Mtb immunity, where the immune system was already “Mtb-imprinted” by a cleared long-term infection. For this purpose we established an Mtb re-infection model by infecting mice with a low dose of 25–50 CFU Mtb Erdman followed by a 12-week antibiotic course of isoniazid (INH) and rifabutin (RIF) starting 4 weeks into the infection (**Figure 3G**). H83 and H89 vaccination was initiated after 10 weeks of antibiotic treatment starting from week 14. After complete antibiotic treatment, mice were challenged with a secondary Mtb infection of 50–100 CFU Mtb Erdman and the bacterial burden was assessed in the lungs 4 and 8 weeks later. At the early time point, there was no difference in protection between H83 and H89 (**Figure 3H**, left). However, similar to the findings in the long-term chronic model, the bacterial burden in H83-vaccinated mice was significantly lower (-Δlog 0.84) than both H89-vaccinated (-Δlog 0.48) and Mtb-memory mice (-Δlog 0.45) compared to age-matched naïve mice at the 8 week time point (**Figure 3H**, right). Additionally, H83 was the only group where bacterial numbers were reduced from week 4 to week 8 (from 4.70 to 4.47), emphasizing that incorporation of ESAT-6 in the vaccine was required for providing additional control on top of an established Mtb memory immune response.

In summary, we demonstrated that a fusion protein of some of the most immunodominant and protective Mtb antigens, H83, was able to induce long-lasting control of both a chronic Mtb infection in naïve mice as well as in a secondary infection in Mtb exposed mice. The phenomenon of sustained protection was entirely dependent on the presence of ESAT-6, as a fusion with Rv1198 as a replacement for ESAT-6 (H89), lost long-term bacterial control, despite providing similar short-term protection.

### Vaccination Rescues Terminal Differentiation of ESAT-6 Specific CD4 T Cells

The proliferative capacity of IL-2 producing CD4 T cells induced by vaccination is important for long-term memory and is dependent on the T cell differentiation status (12, 13). To explore whether differences in CD4 T cell differentiation could be associated to the observed differences in long-term protection between H83 and H89, we characterized CD4 T cell subsets specific to all of five immunodominant antigens during Mtb infection.

**Figure 4:**
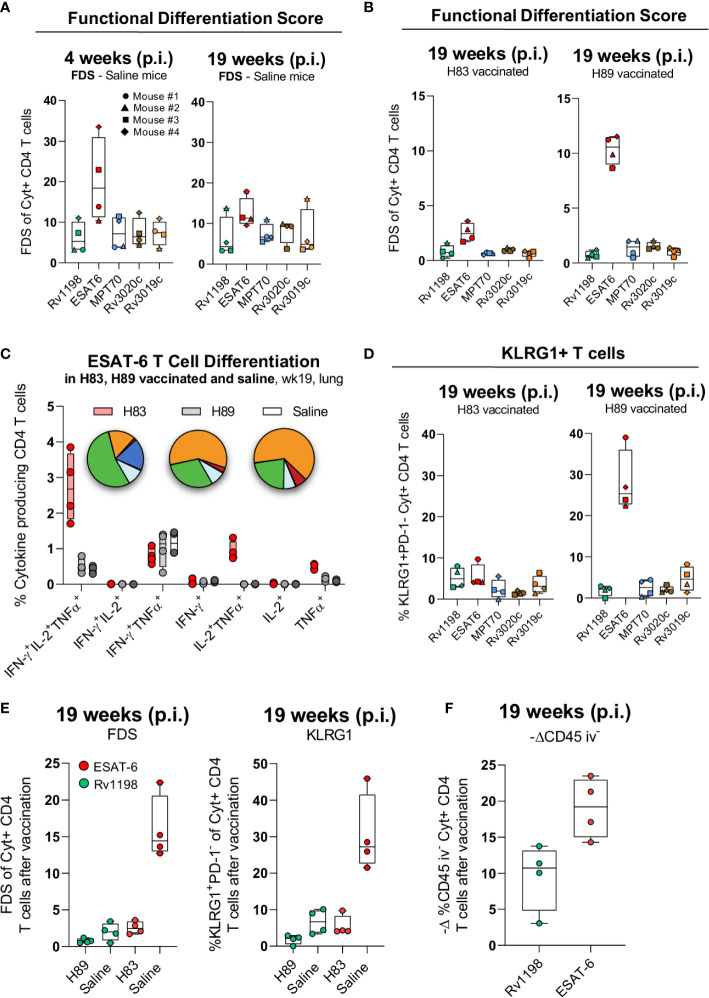
H83 vaccination rescues ESAT-6-specific CD4 T cells from terminal differentiation. **(A)** The Functional Differentiation Score (FDS) of Rv1198, ESAT-6, MPT70, Rv3020c and Rv3019c-specific CD4 T cells from Mtb infected mice at week 4 and 19 after Mtb challenge (n = 4). FDS represents the ratio of all IFN-γ producing CD4 T cell subsets divided by subsets producing other cytokines (IL-2, TNFα), but not IFN-γ (high FDS = high IFN-γ production). **(B)** FDS of Rv1198, ESAT-6, MPT70, Rv3020c, and Rv3019c-specific CD4 T cells from H83 and H89 vaccinated mice at week 4 and 19 after Mtb challenge (n = 4). **(C)** The distribution of cytokines that ESAT-6 specific CD4 T cells produce after H83 (red), H89 (gray), or saline vaccination (white) 19 weeks post Mtb infection (n = 4). Pies nomenclature: green (IFN-γ^+^, IL-2^+^, TNFα^+^), orange (IFN-γ^+^, TNFα^+^), red (IFN-γ^+^), dark blue (IL-2^+^, TNFα^+^), and light blue (TNFα^+^). **(D)** Percentage of KLRG1^+^PD-1^-^ expressing Rv1198, ESAT-6, MPT70, Rv3020c, and Rv3019c-specific CD4 T cells from H83 and H89 vaccinated mice 4 and 19 weeks after Mtb challenge (n = 4). **(E)** (left) FDS and (right) KLRG1^+^PD-1^-^ expressing (right) ESAT-6 and Rv1198-specific CD4 T cells. **(F)** Increase in lung homing capacity of ESAT-6 and Rv1198-specific CD4 T cells after vaccination shown as –delta difference in CD45 iv^-^ staining. The delta graph for CD45 iv^-^ was calculated as the mean value for ESAT-6 and Rv1198 in infected mice subtracted the value for ESAT-6 in H83 vaccinated mice and Rv1198 in H89 vaccinated mice 19 week post infection (n = 4). Graphs are visualized as box plots showing all points with whiskers indicating minimum and maximum values.

The pattern of cytokine expression measured by flow cytometry can be a sensitive indicator of a CD4 T cell’s differentiation state. In this regard, a Functional Differentiation Score (FDS) defined as the ratio of all IFN-γ producing CD4 T cell subsets divided by subsets producing other cytokines (IL-2, TNFα) but not IFN-γ, represents a simple measure of the overall differentiation state of the CD4 T cell repertoire (30, 47). The higher the FDS, the more terminally differentiated T cell repertoire. We initiated the T cell characterization by comparing the FDS of CD4 T cells specific for the five different antigens following natural infection with Mtb. Notably, the ESAT-6-specific CD4 T cells stood out with the highest FDS compared to CD4 T cells against any of the other antigens (**Figure 4A**). Since subunit vaccines are described to induce CD4 T cells of lower differentiation than live mycobacteria (16), we next investigated the impact of vaccination with H83 or H89 on each of the individual antigen-specific CD4 T cell subsets. Generally, vaccination had some impact on lowering FDS for all antigens compared to naïve mice, but except for ESAT-6, there were little differences between H83 and H89 vaccinated animals (**Figure 4B**). In contrast, vaccination with H83 reduced the ESAT-6-specific FDS significantly from 10.6 in H89 vaccinated animals (similar to saline injected animals **Figure 4A**) to 2.5 in H83 vaccinated animals (**Figure 4B**). An analysis of the cytokine expression profile for ESAT-6-specific CD4 T cells in all groups demonstrated that the ESAT-6 containing vaccine, H83, induced orders of magnitude higher proportions of CD4 T cells expressing TNFα and IL-2 alone (dark blue pie slice) or in combination with IFN-γ (green pie slice) (**Figure 4C**). In contrast, ESAT-6 specific CD4 T cells induced by Mtb infection (saline or H89 vaccinated animals), displayed higher proportions of TNFα^+^ IFN-γ^+^ co-producing CD4 T cells (orange pie slice) and IFN-γ single producing CD4 T cells (red pie slice). Notably, the pool of TNFα^+^IL-2^+^-producing ESAT-6 specific T cells, previously described to have central memory phenotype (13), was a unique feature of the H83 group and was completely absent in H89-vaccinated and saline mice (**Figure 4C**). This correlated with a pronounced reduction in KLRG1 expressing ESAT-6-specific CD4 T cells in the H83 group (**Figure 4D**). Overall, vaccination with H83 lowered the total FDS value and frequency of KLRG1-expressing H83-specific CD4 T cells to a higher extent than H89-vaccination compared to saline mice (**Supplementary Figure 3**), which was almost exclusively attributed to marked differences between ESAT-6 and Rv1198 CD4 T cells (**Figure 4E**). Finally, the ability of CD4 T cells to migrate into infected tissues is dependent on their differentiation status (11, 15–18, 42, 48). We measured the lung homing capacity of CD4 T cells by injecting anti-CD45 intravenously prior to euthanization. This intravascular labelling allowed us to differentiate CD4 T cells in the lung vasculature from those in the lung interstitium. The lowered differentiation state of ESAT-6-specific CD4 T cells in the H83 group was also associated with an improved lung homing capacity as more ESAT-6 specific CD4 T cells were found in the lung interstitium after vaccination compared to Rv1198 CD4 T cells (**Figure 4F**).

Collectively, these data show that natural Mtb infection drives a more differentiated CD4 T cell response against ESAT-6 compared to MPT70, Rv3019c, Rv3020c and Rv1198. Vaccination with an ESAT-6 containing vaccine (H83) therefore had a bigger relative impact on T cell differentiation and rescued CD4 T cells from terminally differentiation, possibly explaining the increased long-term protection of H83 compared to H89.

## Discussion

Mtb causes long-term chronic infections and one of the most important requirements for a future TB vaccine is durable protection. However, little is known about the requirements for inducing long-term protection and the role of antigen composition has not been investigated. Most of the subunit vaccine candidates under development incorporate immunodominant antigens and in this study we examined the differential role of a selection of the top immunodominant antigens in two long-term Mtb mouse models.

ESAT-6 is one of the most well characterized Mtb proteins and only few other antigens are used as Mtb-reference in immunological studies. The main goal was therefore to compare ESAT-6 with other less-characterized immunodominant antigens. Out of 62 screened antigens, we identified ESAT-6 along with MPT70, Rv3020c, and Rv3019c as the top four most immunogenic antigens during long-term murine Mtb infection. This is concordant with all of these antigens being ranked as the top 20 most immunodominant and immunogenic *in vivo* expressed TB proteins (IVE-TB) in *Mtb*-exposed individuals (21), that they (or their homologous partners) show 100% CD4 T cell reactivity in individuals with LTBI (37), and that all of them are described as vaccine targets (34, 35, 49). However, the antigens were identified based on screening with *in silico* predicted epitopes of 17 amino acids, and we cannot rule out that other immunodominant antigens would have come up with other peptide lengths. Also, although antigen conservation is a hallmark of Mtb (50), there is some variation in immunodominance between Mtb strains (50, 51). Future studies should therefore address, whether the immune dominant antigens observed for Mtb Erdman are the same in other clinical strains. After identification, we confirmed that each antigen provided robust individual protection in the standard short-term preventive model. Of note, MPT70, Rv3019c, and ESAT-6 were more immunogenic than Rv3020c during natural Mtb infection but this did not translate into vaccine immunogenicity. This complexity emphasizes that antigen hierarchy is context dependent and underscore the need for analyzing individual antigen responses in vaccine studies. Comparable observations in the immune hierarchy have been seen for other vaccines such as ID93, H4, and H56 (26, 52, 53).

We next investigated long-term vaccine protection of the antigens when combined into a fusion protein, H83. In the standard short-term preventive model, H83 induced levels of protection similar to BCG. However, when the infection progressed into the long-term chronic phase, BCG-induced protection was lost while H83 remained significantly protective. This is consistent with a previous study, showing that H56 (containing ESAT-6) and BCG induced similar protection early in the infection phase but 12 and 24 weeks after challenge, H56 was superior to BCG (9). Together these studies highlight that adjuvanted protein vaccines have an advantage over BCG in inducing sustainable protection (54), which might be associated to induction of less differentiated CD4 T cell subsets with increased proliferative potential (e.g. IL-2^+^TNFα^+^) seen in both mice (16) and humans (55, 56).

ESAT-6 has previously been suggested to be essential for improved protection of modified mycobacterial vaccines (27, 57, 58) as well as post-exposure immunity of subunit vaccines (29). Based on this, we asked whether ESAT-6 was essential for the observed long-term protection of H83 or if this was a general trait of highly immunodominant antigens. In the standard short-term model there was no difference in the protective efficacy of H83 and the H89 vaccine where ESAT-6 was replaced by a similar ESAT-6 family member protein, Rv1198 (EsxL). At the late time point (24 weeks post infection), however, the immunity of H89 waned, whereas H83 remained protective as initially observed. This could not be explained by differences in immunogenicity or overall epitope pattern of the vaccines. Importantly, the phenomenon of increased protection from H83 was robust, as we made the same observation in an Mtb re-infection model of post-exposure vaccination. This is in line with previous reports documenting post-exposure protection of ESAT-6 based vaccines in both guinea pigs (59) and mice (60–62), and our results expand on this with new knowledge regarding durability of vaccine-mediated protection in the long-term preventive setting. The unique role of ESAT-6 in both long-term and post-exposure protection could suggest common features in the underlying mechanisms leading to containment of infection and prevention of disease progression. Our observations do not rule out that antigen combinations without ESAT-6 can confer post-exposure efficacy in pre-clinical models, e.g. like with ID93/GLA-SE (44), but highlight that antigen-candidates for future TB vaccines should be screened individually for both post-exposure and long-term preventive protection as a key selection criterion.

The T cell differentiation state dictates both the proliferative capacity/memory potential of CD4 T cells (12, 13), their ability to populate infected lung tissue (10–14) and their metabolic state (63), which all represents important features of long-lived immunity to Mtb. Moguche and colleagues (30) recently reported that CD4 T cells against ESAT-6 are highly differentiated during Mtb infection in mice and humans, and we hypothesized that the unique role of ESAT-6 in long-term protection could be associated to antigenic differences in T cell differentiation state. By characterizing the T cell differentiation in Mtb infected mice, we showed that ESAT-6-specific CD4 T cells had a significantly higher differentiation status measured by both FDS (cytokine expression pattern) and KLRG1 expression compared to the other antigens. To our knowledge, this is the first formal demonstration that antigens selected for high IFN-γ responses during Mtb infection display marked differences in their CD4 T cell quality and functionality, meaning that “immunodominant antigens” and their concomitant T cell responses cannot *a priori* be regarded as “similar”. In line with this, a human study that restimulated donor cells from infected individuals with different Mtb-antigens, demonstrated that there is variation in the cytokine secretion pattern (21, 33) highlighting the importance of addressing antigen-specific heterogeneity in immunological and vaccine studies. This heterogeneity might be associated to differential *in vivo* antigen expression (30, 64) although the currently available studies are inconclusive on this aspect (46).

Since adjuvanted subunit vaccines have been shown to induce CD4 T cells of lower differentiation compared to mycobacterial infection (13, 16), we next explored the impact of vaccination. Given that ESAT-6-specific CD4 T cells were particularly differentiated during Mtb infection, we hypothesized that vaccination with an ESAT-6-containing vaccine (H83) would lead to a greater relative reduction in overall T cell differentiation compared to a non-ESAT-6 vaccine (H89). Both H83 and H89 had a positive impact on T cell differentiation, but we found the most dramatic improvement in T cell differentiation state for H83, which primarily was attributed to ESAT-6 responses. Thus, during long-term Mtb infection, ESAT-6-specific CD4 T cells in H83-vaccinated animals displayed a substantial decrease in both FDS and KLRG1 expression as well as a bigger relative improvement in the capacity to enter the infected lung tissue. This demonstrates that subunit vaccination has the potential to improve long-term bacterial control by rescuing certain “vulnerable” CD4 T cell populations from terminal differentiation. This is in line with a previous report, showing that improved long-term immunity of the H56 subunit vaccine was linked to decreased differentiation of ESAT-6-specific CD4 T cells compared to Mtb/BCG memory immunity (16) as well as a study demonstrating that improved post-exposure protection by low dose vaccination was associated with induction of less differentiated IL-2 producing CD4 T cells (65). However, our results do not exclude that there are other potential contributors to the seemingly unique vaccine properties of ESAT-6. This could e.g. include antibodies blocking ESAT-6-mediated virulence or variations in local antigen concentration/positioning during infection, where effector CD4 T cells would need to recognize Mtb infected cells instead of bystander cells presenting soluble secreted antigens. Regarding the latter, some Mtb proteins have already been described as decoys antigens (33, 66).

Overall this study highlights the need for long-term readouts in both preclinical and clinical studies. In addition to the recent clinical trials demonstrating signals of vaccine efficacy of two subunit vaccines (2, 4), the observation that the H83 vaccine remained completely protective when BCG immunity waned further supports the subunit platform for TB vaccine development (54). So far, most TB vaccine discovery approaches have relied on IFN-γ as the leading biomarker for antigen identification, but our data underlines that antigens with similar response magnitude should not be regarded equally relevant for long-term protection. Instead we suggest that T cell differentiation (e.g. measured by FDS) could represent a more precise measurement of “immunodominance” and future efforts in antigen discovery should include a focus on identifying antigens with high T cell differentiation, like ESAT-6. Vaccination with a combination of such antigens would likely have the biggest impact on preventing terminal differentiation of CD4 T cells and potentially maximize long-term protective efficacy. Some of the vaccine candidates that have been or are in clinical trials include ESAT-6 and other immunodominant antigens (2, 24–26, 28, 53), and data from these trials, as well as detailed studies of infection-driven immune responses in humans, should be able to address whether the differences found in our animal models are also true in the human setting.

## Data Availability Statement

The raw data supporting the conclusions of this article will be made available by the authors, without undue reservation.

## Ethics Statement

The animal studies were reviewed and approved by Statens Serum Institut’s Animal Care and Use Committee headed by DVM, Kristin Engelhart and DVM, Louise Kragh Isling.

## Author Contributions

HC, RM, CA, and PA conceived and designed the studies. HC, NK, and RB performed murine TB experiments and analyzed the data. IR and CA designed the recombinant proteins. HC and RM drafted the manuscript. HC, GJ, PA, and RM finalized the manuscript. All authors contributed to the article and approved the submitted version.

## Funding

This work was supported by the Lundbeck Foundation (R249-2017-851), the Independent Research Fund Denmark (DFF – 7025-00106, DFF - 7016-00310) and the National Institutes of Health/National Institute of Allergy and Infectious Diseases (Grant 1R01AI135721-01).

## Conflict of Interest

PA, CA, and RM are co-inventors of patents covering ESAT-6 containing vaccines other than H83 of this study that was designed for scientific purposes. PA and IR are also co-inventors of patents covering the use of CAF01 as an adjuvant. 

The remaining authors declare that the research was conducted in the absence of any commercial or financial relationships that could be construed as a potential conflict of interest.

## Supplementary Material

Supplementary Table 1 Human recognized peptides screened in the CB6F1 during chronic Mtb infection. Female CB6F1 mice were challenged with Mtb Erdman by the aerosol route. At late-stage chronic Mtb infection (wk20-24), splenocytes were harvested and *in vitro* stimulated with a range of 318 Mtb-derived peptides for 72 h. The supernatants were analyzed for IFN-γ levels. The peptide sequence and exact IFN-γ levels from two independent experiments are indicated. The four immunodominant antigens identified in **Figure 1B** is highlighted in the table in bold and colored text.

Supplementary Figure 1 Gating strategy for antigen-specific CD4 T cells. Single-cell suspensions were prepared from the spleens or lungs of vaccinated or infected mice. Single cells were initially gated from a FSC-A and FSC-H plot. Lymphocytes were afterwards gated from a SSC-A and FSC-A plot. Non-viable cells were excluded by staining with fixable eflour-780 dye. CD3 and CD4 double positive T cells were gated followed by a gate for CD44^high^ and TNFα, IL-2 and IFN-γ positive CD4 T cells. Boolean gating was performed on all samples, combining CD4 T cells producing either TNFα, IL-2 or IFN-γ into one group (antigen-specific CD4 T cells). Antigen-specific CD4 T cells were further characterized for their expression of KLRG1 and PD-1 and CD45. Only the KLRG1^+^PD-1^-^ subset is visualized in the corresponding figures. Combination gates for TNFα, IL-2, or IFN-γ producing CD4 T cells were created and the T cell differentiation state expressed as the functional differentiation score (FDS). FDS represents the ratio of all IFN-γ producing CD4 T cell subsets divided by subsets producing other cytokines (IL-2, TNFα), but not IFN-γ (high FDS = high IFN-γ production). Fluorescence minus one controls for KLRG1 and PD-1 are shown.

Supplementary Figure 2 Epitope recognition pattern in H83 and H89 vaccinated mice. CB6F1 mice were immunized three times with CAF01 adjuvant alone, 0.5 µg recombinant H83 or H89. Six weeks after third immunization, mice were challenged with Mtb Erdman. Four weeks post Mtb infection, lung cells from two mice were harvested and pooled within each group. Technical duplicates of pooled lungs cells were *in vitro* restimulated using 16-mer peptides with 11 amino acid overlap spanning all five antigens comprising H83 and H89. As control, lung cells were stimulated with media only (None) or phorbol 12-myristate 13-acetate and ionomycin (PMA-Iono). Culture supernatants were harvested after 3 days of culture and analyzed for levels of IFN-γ by ELISA. Epitopes with the highest responses are indicated with the peptide sequence used for stimulation.

Supplementary Figure 3 H83-vaccinated mice display a greater reduction in FDS and KLRG1-expressing CD4 T cells after vaccination. The difference in functional differentiation score (FDS) (left) and KLRG1 expression (right) of H83- and H89-specific CD4 T cells in respectively H83- and H89-vaccinated mice compared to saline mice (n = 4). FDS represents the ratio of all IFN-γ producing CD4 T cell subsets divided by subsets producing other cytokines (IL-2, TNFα), but not IFN-γ (high FDS = high IFN-γ production). Delta FDS values and KLRG1^+^PD-1^-^ were calculated as the mean value for H83 and H89-specific CD4 T cells in infected mice subtracted the value for H83-specific CD4 T cells in H83 vaccinated mice and H89-specific CD4 T cells in H89 vaccinated mice 19 week post infection. Graphs are visualized as box plots showing all points with whiskers indicating minimum and maximum values.
