# Supplementary material for: Rescuing ESAT-6 Specific CD4 T Cells From Terminal Differentiation Is Critical for Long-Term Control of Murine Mtb Infection
Source: Front Immunol. 2020 Nov 6;11:585359. doi: 10.3389/fimmu.2020.585359 (PMC7677256; doi:10.3389/fimmu.2020.585359)
Supplement: Supplementary Table 1 — Human recognized peptides screened in the CB6F1 during chronic Mtb infection. Female CB6F1 mice were challenged with Mtb Erdman by the aerosol route. At late-stage chronic Mtb infection (wk20-24), splenocytes were harvested and in vitro stimulated with a range of 318 Mtb-derived peptides for 72 h. The supernatants were analyzed for IFN-γ levels. The peptide sequence and exact IFN-γ levels from two independent experiments are indicated. The four immunodominant antigens identified in Figure 1B is highlighted in the table in bold and colored text. [file Table_1.docx]

**Supplementary Table 1.**

| Experiment #1 wk24 post infection | | | Experiment #2 wk20 post infection | | |
| --- | --- | --- | --- | --- | --- |
| **Rv gene annotation** | **Peptide Sequence**  ***17-mers*** | ***IFN-y (pg/*ml)** | **Rv gene annotation** | **Peptide Sequence**  ***17-mers*** | ***IFN-y (pg/*ml)** |
| Rv3875_01 | TEQQWNFAGIEAAASAI | 12935.6 | *TB10.4* | ASDLFSAASAFQSVVWG | 19579.7 |
| Rv3019c_01 | SQIMYNYPAMMAHAGDM | 7199.0 | *Rv2875_04* | GCAEYAAANPTGPASVQ | 9772.5 |
| Rv2875_04 | GCAEYAAANPTGPASVQ | 7153.4 | *Rv3875_01* | TEQQWNFAGIEAAASAI | 7663.2 |
| Rv2875_03 | GPGCAEYAAANPTGPAS | 6037.1 | *Rv3019c_01* | SQIMYNYPAMMAHAGDM | 7157.2 |
| Rv2892c_04 | AAAAYELAFAMTVPPPV | 5729.0 | *Rv2875_03* | GPGCAEYAAANPTGPAS | 6672.7 |
| Rv2892c_03 | GQAEQAGMQARAAAAAY | 5122.7 | *Rv3874_01* | LQGQWRGAAGTAAQAAV | 4448.3 |
| Rv3478_07 | LHSMLKGLAPAAAQAVE | 2564.2 | *Rv3020c_01* | GQAEQQAMSAQAFHQGE | 4176.7 |
| Rv3020c_01 | GQAEQQAMSAQAFHQGE | 2459.7 | *Rv3873_02* | SDKALAAATPMVVWLQT | 3506.1 |
| Rv3136_03 | EKTQQTAIQARAAALAF | 2038.4 | *Rv3136_03* | EKTQQTAIQARAAALAF | 2829.7 |
| Rv3874_01 | LQGQWRGAAGTAAQAAV | 1727.1 | *Rv3478_07* | LHSMLKGLAPAAAQAVE | 2076.7 |
| Rv3018c_01 | TAPVWLASPPEVHSALL | 1690.9 | *Rv3020c_03* | AHARFVAAAAKVNTLLD | 1946.9 |
| Rv0287_03 | AHARFVAAAAKVNTLLD | 1578.2 | *Rv2875_05* | SGQYTVFAPTNAAFSKL | 1867.2 |
| Rv3125c_01 | MVLGFSWLPPEINSARM | 1574.6 | *Rv1387_05* | PMLAVAGVGPAVAAPGM | 1641.8 |
| Rv3621c_03 | AAAMYGYAGASAAATQL | 1429.9 | *Rv3135_01* | MDYAFLPPEINSARMYS | 1453.9 |
| Rv3020c_03 | AHARFVAAAAKVNTLLD | 1400.9 | *Rv3135_02* | INSARMYSGPGPNSMLV | 1354.9 |
| Rv3136_07 | AMYGYATASAAAALLTP | 1380.7 | *Rv1387_04* | APPMLAVAGVGPAVAAP | 1331.7 |
| Rv3135_01 | MDYAFLPPEINSARMYS | 1233.5 | *Rv3478_08* | MLKGLAPAAAQAVETAA | 1281.9 |
| Rv2875_05 | SGQYTVFAPTNAAFSKL | 1195.0 | *Rv3125c_02* | SGPLFAAASAWEGLAAD | 1265.6 |
| Rv1317c_05 | AAARFESATASAGTVSL | 1132.6 | *Rv0287_03* | AHARFVAAAAKVNTLLD | 1254.5 |
| Rv3125c_02 | SGPLFAAASAWEGLAAD | 1117.8 | *Rv0442c_05* | AAAQAEQAAAQAMAIAT | 1251.5 |
| Rv3021c/  Rv3022c_01 | TAPVWLASPPEVHSALL | 1076.5 | *Rv3125c_01* | MVLGFSWLPPEINSARM | 1162.6 |
| Rv2608_01 | AASAFEATLAATVSPAM | 1032.3 | *Rv0256c_10* | GLAGLVGIPPSAPPVIP | 1142.4 |
| Rv2892c_01 | SGRMYAGPGSGPMMAAA | 1025.4 | *Rv1387_03* | ELTPVAAAPPMLAVAGV | 1100.5 |
| Rv1387_05 | PMLAVAGVGPAVAAPGM | 1012.0 | *Rv3018c_02* | LVSAVEPAPASTSVSVL | 1096.0 |
| Rv1802_10 | AMYGYAGSSSVATQVTP | 994.4 | *Rv2892c_01* | SGRMYAGPGSGPMMAAA | 1040.1 |
| Rv3330_02 | IGLFYRYAWQNPVFADI | 935.9 | *Rv3478_04* | YETAYRLTVPPPVIAEN | 994.1 |
| Rv3478_09 | LRVPARAYAIPRTPAAG | 913.9 | *Rv1172c_02* | NAANVVAAVPTTGVLAA | 960.1 |
| Rv0442c_01 | TSPHFAWLPPEINSALM | 912.0 | *Rv3478_06* | AMYGYAATAATATEALL | 932.4 |
| Rv3621c_01 | VNSALMYAGPGSGPMLA | 893.5 | *Rv3876_05* | VINHIMPGEPNVAVKDL | 909.3 |
| Rv0287_02 | SSAAFQAAHARFVAAAA | 892.3 | *Rv3621c_03* | AAAMYGYAGASAAATQL | 901.9 |
| Rv3873_03 | QAAAYTQAMATTPSLPE | 852.8 | *Rv2875_02* | AALAVAVSPPAAAGDLV | 854.8 |
| Rv0287_04 | AAGTYVAADAAAASTYT | 850.6 | *Rv0256c_07* | VSTAAVAAAPQTTPAPQ | 842.7 |
| Rv3478_08 | MLKGLAPAAAQAVETAA | 843.5 | *Rv0287_04* | AAGTYVAADAAAASTYT | 840.7 |
| Rv0453_01 | SALIWMASPPEVHSALL | 830.8 | *Rv2608_02* | AMFGYHSAASAVATQLA | 829.7 |
| Rv0280_20 | QPDSAAAAAAAAAVRDQ | 830.1 | *Rv3020c_04* | AAGTYVAADAAAASSYT | 824.3 |
| Rv1196_08 | AMFGYAAATATATATLL | 808.5 | *Rv3136_04* | AALAFEQAYAMTLPPPV | 814.9 |
| Rv0256c_01 | TAPIWMASPPEVHSALL | 807.9 | *Rv3804c_03* | HPQQFVYAGAMSGLLDP | 793.7 |
| Rv2892c_05 | YELAFAMTVPPPVVVAN | 806.4 | *Rv3621c_04* | AMYGYAGASAAATQLSP | 788.4 |
| Rv1172c_08 | WIRGFLPPLPPISPPGF | 804.0 | *Rv2608_01* | AASAFEATLAATVSPAM | 739.8 |
| Rv1800_03 | MAGYYVGASAVATQLAS | 801.7 | *Rv1808_02* | YETAFAATVPPPVIEAN | 719.7 |
| Rv2608_02 | AMFGYHSAASAVATQLA | 788.5 | *Rv1361c_04* | GQAELTAAQVRVAAAAY | 692.0 |
| Rv0280_01 | MTLWMASPPEVHSALLS | 775.1 | *Rv2608_03* | PQLGFTLSGATPADAYP | 674.1 |
| Rv1172c_04 | ALLPRAGAAAAAALPAL | 764.8 | *Rv3021c/ Rv3022c_01* | TAPVWLASPPEVHSALL | 672.9 |
| Rv3621c_04 | AMYGYAGASAAATQLSP | 753.7 | *Rv1808_04* | AMYGYAGSSATASQLAP | 667.2 |
| Rv3478_03 | GQAQLTAAQVRVAAAAY | 753.5 | *Rv0286_01* | AAPIWMASPPEVHSALL | 636.5 |
| Rv1802_09 | AAAMYGYAGSSSVATQV | 747.1 | *Rv2608_05* | SGLGLPPPWQPALPRLF | 634.5 |
| Rv1802_13 | MSVPPSWAAATPAIRTV | 735.6 | *Rv3136_07* | AMYGYATASAAAALLTP | 634.4 |
| Rv1387_01 | EMAAQQHVVIEAYTAAV | 726.8 | *Rv3478_05* | AEAMYGYAATAATATEA | 628.6 |
| Rv1808_03 | AAAMYGYAGSSATASQL | 720.7 | *Rv3478_02* | ASDLFSAASAFQSVVWG | 613.0 |
| Rv2875_02 | AALAVAVSPPAAAGDLV | 719.2 | *Rv3025c_01* | ATTPMHPAAIEAMAAVQ | 605.7 |
| Rv2490c_01 | HAQEYQALSAQAAAFHD | 716.6 | *Rv0280_01* | MTLWMASPPEVHSALLS | 602.0 |
| Rv3025c_01 | ATTPMHPAAIEAMAAVQ | 693.1 | *Rv3330_02* | IGLFYRYAWQNPVFADI | 600.4 |
| Rv3135_03 | SARMYSGPGPNSMLVAA | 691.9 | *Rv3621c_02* | AASMVAAATPQVAWLRS | 594.0 |
| Rv3136_05 | FEQAYAMTLPPPVVAAN | 691.3 | *Rv3873_05* | TRPGLVAPAPLAQEREE | 593.7 |
| Rv3024c_01 | QQIKFAALSARAVALGF | 689.8 | *Rv3478_03* | GQAQLTAAQVRVAAAAY | 588.8 |
| Rv3018c_02 | LVSAVEPAPASTSVSVL | 685.2 | *Rv0286_11* | PSAGAAPAPAAPATASF | 587.8 |
| Rv0291_02 | YAAGYVSGVAALVRSRY | 684.5 | *Rv3621c_01* | VNSALMYAGPGSGPMLA | 584.0 |
| Rv1199c /  Rv2512c | YAANLMAATPKPSWPWV | 683.9 | *Rv0256c_06* | YQAVSTAAVAAAPQTTP | 583.1 |
| Rv3136_04 | AALAFEQAYAMTLPPPV | 682.4 | *Rv1808_03* | AAAMYGYAGSSATASQL | 580.7 |
| Rv0987_01 | IFAAMLATLPPAIEAMR | 679.7 | *Rv0286_13* | GRGGIKAPAATVPAAAA | 575.3 |
| Rv1705c_05 | AMYGYAGSSATATKVTP | 678.5 | *Rv3330_01* | AACPYKVSTPPAVDSSE | 574.1 |
| Rv0256c_04 | NESDYARMWIQAATTMA | 673.3 | *Rv1802_13* | MSVPPSWAAATPAIRTV | 569.5 |
| Rv2892c_11 | SWGPTIFAGPRASPSVA | 669.3 | *Rv0280_07* | MQPAAIPALAPVAAAPS | 563.8 |
| Rv0280_09 | PALAPVAAAPSTLPAVA | 666.4 | *Rv0442c_01* | TSPHFAWLPPEINSALM | 557.3 |
| Rv1366_01 | EYPGQRAMALGEASKNK | 665.5 | *Rv0291_01* | VAPAARLLSIRAMSTKF | 553.2 |
| Rv1706c_04 | FEAAFAMTVPPPAIAAN | 658.2 | *Rv3876_02* | PSASFPPAPASANLPKP | 552.7 |
| Rv3020c_02 | SAAAFQGAHARFVAAAA | 656.9 | *Rv0286_09* | ASGAALASAPRTVPAPT | 552.3 |
| Rv0279c_01 | MSFVIAAPEVIAAAATD | 656.6 | *Rv3330_04* | NQLLYNYPGALGGKTGY | 551.2 |
| Rv0286_01 | AAPIWMASPPEVHSALL | 656.3 | *Rv1196_08* | AMFGYAAATATATATLL | 550.7 |
| Rv3125c_06 | VASQAQLAAIQARAAAT | 649.2 | *Rv3125c_06* | VASQAQLAAIQARAAAT | 550.5 |
| Rv1800_02 | AVSVFEEALAATVHPAM | 647.6 | *Rv2490c_01* | HAQEYQALSAQAAAFHD | 546.3 |
| Rv2608_03 | PQLGFTLSGATPADAYP | 645.3 | *Rv1802_14* | EGSLLSQMALASVAGGA | 543.3 |
| Rv1172c_03 | SADAYATAEASAAQTMV | 642.9 | *Rv0286_02* | AVPGWAWQGPSAEWYVA | 540.6 |
| Rv3621c_02 | AASMVAAATPQVAWLRS | 641.0 | *Rv2892c_10* | GLLQFEASLAQQAIPGT | 540.5 |
| Rv1705c_04 | AMAMYGYAGSSATATKV | 638.5 | *Rv3135_03* | SARMYSGPGPNSMLVAA | 529.9 |
| Rv1196_06 | TAYGLTVPPPVIAENRA | 637.7 | *Rv3876_01* | AQPFFDPSASFPPAPAS | 527.6 |
| Rv0453_02 | RLEAVTAAYAAALVAMP | 632.2 | *Rv3621c_05* | ALPAAVPAIPSAGLSGV | 527.2 |
| Rv0442c_05 | AAAQAEQAAAQAMAIAT | 632.0 | *Rv0256c_09* | PLAPLTLAPASAAGGFA | 525.1 |
| Rv0286_07 | VMGLYQAASGAALASAP | 631.7 | *Rv2853_01* | FHSRFVQALTTAAASYA | 524.2 |
| Rv3873_02 | SDKALAAATPMVVWLQT | 629.6 | *None* | INSARMYAGPGSASLVA | 521.0 |
| Rv3873_01 | LNTARLMAGAGPAPMLA | 629.4 | *Rv2823c_02* | RLFIATGSVPLAANDLM | 515.0 |
| Rv3024c_02 | GDTPKRQIRAEAARRGL | 627.1 | *Rv0256c_08* | APQIVKANAPTAASDEP | 513.3 |
| Rv0442c_06 | EQAAAQAMAIATAFEAA | 621.8 | *Rv1789_03* | PYVAWMSAAAAQAEQAA | 510.9 |
| Rv3020c_04 | AAGTYVAADAAAASSYT | 618.5 | *Rv2892c_02* | ELTGAYWAGPAAASMVA | 510.3 |
| Rv1706c_03 | AAAAFEAAFAMTVPPPA | 616.8 | *Rv3478_01* | INSARMYAGPGSASLVA | 508.5 |
| Rv3125c_03 | PWTGPASMSMAAAASPY | 615.4 | *Rv3478_09* | LRVPARAYAIPRTPAAG | 505.2 |
| Rv1705c_02 | AAAAYETAFAAIVPPPL | 613.9 | *Rv2892c_03* | GQAEQAGMQARAAAAAY | 502.8 |
| Rv3330_04 | NQLLYNYPGALGGKTGY | 610.1 | *Rv0256c_05* | ARMWIQAATTMASYQAV | 501.8 |
| Rv1789_03 | PYVAWMSAAAAQAEQAA | 608.5 | *Rv1802_07* | YEAAFAATVPPPVVAAN | 499.1 |
| Rv1802_01 | SGRMYAGPGSGPMLAAA | 608.2 | *Rv0256c_11* | PVAAIAPSIPTPTPTPA | 498.7 |
| Rv0129c_01 | IKVQFQGGGPHAVYLLD | 607.7 | *Rv1196_02* | ASDLFSAASAFQSVVWG | 494.9 |
| Rv0286_05 | ADYVRMWLQAAAVMGLY | 606.5 | *Rv3330_03* | LFYRYAWQNPVFADIVA | 492.5 |
| Rv1441c_01 | SVGSYAAAEAANASPLQ | 604.6 | *Rv0279c_04* | GRDGFNAPASTPLHTLQ | 481.2 |
| Rv3125c_10 | QVGTQVAGMATTASAAV | 596.4 | *Rv0286_05* | ADYVRMWLQAAAVMGLY | 477.3 |
| Rv0256c_03 | AYGTALAAMPTLAELGA | 594.3 | *Rv0280_17* | GAAGFGYPYAIAPPGIG | 474.4 |
| Rv0129c_02 | QQFPYAASLSGFLNPSE | 594.1 | *Rv0280_03* | VAAAYTTAVAAMPTLVE | 471.9 |
| Rv0280_05 | YQAVAEAAVASAPQTTP | 593.8 | *Rv1706c_06* | AAAMYGYASAAAPATVL | 470.8 |
| Rv3125c_04 | LSTVASQAQLAAIQARA | 593.4 | *Rv3136_05* | FEQAYAMTLPPPVVAAN | 467.2 |
| Rv1047 Rv3023c Rv3115_01 | YAANLMAATPKPSWPWV | 592.7 | *Rv0442c_10* | LTNTPVAAPASAPVGGL | 466.1 |
| Rv1800_04 | PAYGYSTSPPNVATPFG | 591.6 | *Rv3804c_01* | IKVQFQSGGANSPALYL | 463.4 |
| Rv3125c_09 | TLAPFSLPPVSLAGLAA | 591.4 | *Rv3125c_05* | TVASQAQLAAIQARAAA | 460.7 |
| Rv1802_14 | EGSLLSQMALASVAGGA | 589.8 | *Rv0286_12* | APAPAAPATASFAYAVG | 459.9 |
| Rv3330_01 | AACPYKVSTPPAVDSSE | 588.8 | *Rv0442c_09* | SSAGYATGGMSTAALSS | 458.2 |
| Rv0286_08 | YQAASGAALASAPRTVP | 588.1 | *Rv2892c_05* | YELAFAMTVPPPVVVAN | 456.9 |
| Rv0280_04 | AYTTAVAAMPTLVELAA | 580.9 | *Rv2123_02* | THAATVATAAAAAHETA | 454.4 |
| Rv0286_09 | ASGAALASAPRTVPAPT | 577.9 | *Rv1361c_05* | YETAYGLTVPPPVIAEN | 453.9 |
| Rv3135_04 | AAAAFEQAHAMTVPPAL | 577.7 | *Rv0279c_02* | LESSIAAANAAAAANTT | 446.1 |
| Rv1706c_07 | AMYGYASAAAPATVLTP | 577.0 | *Rv0256c_01* | TAPIWMASPPEVHSALL | 444.4 |
| Rv1706c_02 | TLAEQAAMQARAAAAAF | 575.8 | *Rv1196_03* | AGLMVAAASPYVAWMSV | 439.4 |
| Rv2873_01 | GGEYTVFAPTNAAFDKL | 571.8 | *Rv0129c_03* | IQHVLNGATPPAAPAAP | 437.1 |
| Rv0256c_02 | AATAYGTALAAMPTLAE | 570.0 | *Rv0280_12* | PAVAMAPTMAAPGAAVA | 435.8 |
| Rv3876_01 | AQPFFDPSASFPPAPAS | 569.9 | *Rv1802_12* | FQKFFNPVTPFNPDLIP | 435.4 |
| Rv0286_03 | AAAAYTTALAAMPTLAE | 569.6 | *Rv3125c_07* | SQAQLAAIQARAAATAF | 432.4 |
| Rv0280_14 | APGAAVASAAAPASAPA | 569.3 | *Rv2123_05* | PLIYFGPFAPLTSPVLL | 432.2 |
| Rv0286_02 | AVPGWAWQGPSAEWYVA | 567.7 | *Rv0280_11* | TLPAVAMAPTMAAPGAA | 430.1 |
| Rv3135_02 | INSARMYSGPGPNSMLV | 566.5 | *Rv0442c_08* | AGYHFDASAAVAQLAPW | 424.9 |
| Rv2892c_10 | GLLQFEASLAQQAIPGT | 565.8 | *Rv0987_01* | IFAAMLATLPPAIEAMR | 419.6 |
| Rv1706c_06 | AAAMYGYASAAAPATVL | 563.6 | *Rv3804c_02* | YSDWYQPACGKAGCQTY | 418.4 |
| Rv1706c_05 | AFAMTVPPPAIAANRTL | 555.9 | *Rv0280_13* | MAPTMAAPGAAVASAAA | 417.2 |
| Rv1705c_01 | YVAWMRATAIQAEQAAS | 554.6 | *Rv0280_06* | GLAGMQPAAIPALAPVA | 415.8 |
| Rv3330_03 | LFYRYAWQNPVFADIVA | 553.8 | *Rv1199c /  Rv2512c* | YAANLMAATPKPSWPWV | 414.6 |
| Rv3873_05 | TRPGLVAPAPLAQEREE | 552.7 | *Rv1802_05* | REAAAQASAAAAAYEAA | 413.8 |
| Rv0129c_03 | IQHVLNGATPPAAPAAP | 552.6 | *Rv1047 Rv3023c Rv3115_01* | YAANLMAATPKPSWPWV | 409.8 |
| Rv0279c_03 | GGGAYAAAEAAATSPLL | 551.1 | *Rv2892c_04* | AAAAYELAFAMTVPPPV | 409.1 |
| Rv0286_06 | RMWLQAAAVMGLYQAAS | 549.5 | *Rv1802_08* | VAANRAELAVLAATNIF | 408.3 |
| Rv1387_06 | VGPAVAAPGMLPASAPA | 546.3 | *Rv3024c_02* | GDTPKRQIRAEAARRGL | 407.3 |
| Rv1802_02 | AAPYVAWMSATAALARE | 545.9 | *Rv1361c_01* | INSARMYAGPGSASLVA | 406.3 |
| Rv1789_07 | AMYAYAGSSASASAVTP | 543.5 | *Rv1172c_01* | MSFVFAAPEALAAAAAD | 405.3 |
| Rv1172c_05 | PRAGAAAAAALPALAAE | 533.2 | *Rv1196_05* | YETAYGLTVPPPVIAEN | 404.9 |
| Rv3125c_07 | SQAQLAAIQARAAATAF | 533.1 | *Rv3125c_03* | PWTGPASMSMAAAASPY | 403.7 |
| Rv0297_02 | HAQAYQAASAQAAAFHA | 529.6 | *Rv1808_05* | ATAAQSAVVAQAAGAAA | 403.4 |
| Rv3125c_11 | TQVAGMATTASAAVTPV | 526.2 | *Rv1802_09* | AAAMYGYAGSSSVATQV | 402.7 |
| Rv1361c_05 | YETAYGLTVPPPVIAEN | 523.5 | *Rv0292_01* | RTTVLVWVGPPASDTNV | 402.4 |
| Rv0291_01 | VAPAARLLSIRAMSTKF | 521.9 | *Rv3125c_08* | AATAFEAALAATVHPTA | 402.1 |
| Rv1705c_06 | TATKVTPFAPPPNTTSP | 518.7 | *Rv0442c_04* | TSGAWLGPSAAAMMAVA | 401.4 |
| Rv2892c_08 | ELTPFTAAPVTTSPAAL | 516.5 | *Rv0987_02* | RSLSRTSIAIAALMMAV | 401.2 |
| Rv0280_11 | TLPAVAMAPTMAAPGAA | 516.4 | *Rv1706c_03* | AAAAFEAAFAMTVPPPA | 400.4 |
| Rv3024c_03 | RAPVFTAGAAPSGPVDC | 513.7 | *Rv0297_03* | GGAIFNAGTPGAAGTGG | 399.1 |
| Rv0286_13 | GRGGIKAPAATVPAAAA | 512.8 | *Rv1802_01* | SGRMYAGPGSGPMLAAA | 399.0 |
| Rv1361c_04 | GQAELTAAQVRVAAAAY | 512.7 | *Rv0442c_06* | EQAAAQAMAIATAFEAA | 397.4 |
| Rv2123_04 | VLDPLIYFGPFAPLTSP | 505.3 | *Rv1317c_04* | LARAQRMQTARVLIETT | 396.6 |
| Rv1361c_03 | AGLMVAAASPYVAWMSV | 502.0 | *Rv2874_03* | ILAAIVVAGATATIGLG | 395.4 |
| Rv3136_08 | VPPSWAAPSTRPVSALS | 501.8 | *Rv1196_06* | TAYGLTVPPPVIAENRA | 395.0 |
| Rv0290_02 | LVLQPVPAGPAAPGIVE | 501.6 | *Rv1172c_07* | AGAAAAAALPALAAESI | 394.4 |
| Rv0293c_01 | GLSGFAEAMAQAVYDFR | 501.0 | *Rv3876_03* | LSERFVSAPPPPPPPPP | 394.2 |
| Rv1705c_07 | KVTPFAPPPNTTSPSAA | 497.6 | *Rv2892c_07* | AMYAYAGSAAIATELTP | 391.8 |
| Rv0280_03 | VAAAYTTAVAAMPTLVE | 497.5 | *Rv3873_03* | QAAAYTQAMATTPSLPE | 391.6 |
| Rv1387_03 | ELTPVAAAPPMLAVAGV | 497.1 | *Rv1196_10* | MLKGFAPAAAAQAVQTA | 384.5 |
| Rv1789_02 | VAPYVAWMSAAAAQAEQ | 497.1 | *Rv0453_02* | RLEAVTAAYAAALVAMP | 383.9 |
| Rv1705c_03 | YETAFAAIVPPPLIAAN | 496.8 | *Rv2823c_01* | MFNRFGSGTANLAFAPE | 383.5 |
| Rv1196_01 | INSARMYAGPGSASLVA | 496.4 | *Rv0280_02* | QTGAWQGPSAAAYVAAH | 380.8 |
| Rv3136_06 | AAAMYGYATASAAAALL | 495.0 | *Rv0442c_02* | SALMFAGPGSGPLIAAA | 378.4 |
| Rv3478_05 | AEAMYGYAATAATATEA | 494.1 | *Rv3021c/ Rv3022c_02* | LVSAVEPAPASTSVSVL | 377.5 |
| Rv1361c_01 | INSARMYAGPGSASLVA | 493.4 | *Rv1196_01* | INSARMYAGPGSASLVA | 376.0 |
| Rv1789_12 | LSVPPVWSGPLPGSVTP | 488.2 | *Rv1317c_02* | LLQAVVGAGPLALARAQ | 375.8 |
| Rv1791_01 | HAQMYQTVSAQAAAIHE | 487.7 | *Rv1172c_08* | WIRGFLPPLPPISPPGF | 375.0 |
| Rv1196_07 | AAAMFGYAAATATATAT | 486.7 | *Rv2123_03* | VAHESVAATPSTPPAPQ | 375.0 |
| Rv1387_07 | AAPGMLPASAPAPAAAA | 486.6 | *Rv2873_01* | GGEYTVFAPTNAAFDKL | 371.0 |
| Rv1361c_02 | ASDLFSAASAFQSVVWG | 486.4 | *Rv2608_04* | KYPLNVFATANAIAGIL | 367.6 |
| Rv0453_04 | NPQLLVAALTPAISGLG | 484.7 | *Rv0286_03* | AAAAYTTALAAMPTLAE | 366.5 |
| Rv3478_06 | AMYGYAATAATATEALL | 484.3 | *Rv0442c_07* | IATAFEAALAATVQPAV | 362.5 |
| Rv0453_03 | VTAAYAAALVAMPTLAE | 481.1 | *Rv0280_16* | AAVASAAAPASAPAAST | 360.3 |
| Rv0442c_04 | TSGAWLGPSAAAMMAVA | 479.3 | *Rv0291_04* | RNVAFAGAAALSVLVGL | 359.7 |
| Rv2608_05 | SGLGLPPPWQPALPRLF | 476.4 | *Rv3024c_01* | QQIKFAALSARAVALGF | 359.4 |
| Rv1387_08 | MLPASAPAPAAAAGATA | 471.1 | *Rv1800_01* | SARVFAGAGSAPMLAAA | 358.6 |
| Rv1196_10 | MLKGFAPAAAAQAVQTA | 470.6 | *Rv3136_08* | VPPSWAAPSTRPVSALS | 357.4 |
| Rv3873_04 | RMWNQAALAMEVYQAET | 469.7 | *Rv1361c_10* | MLKGFAPAAAQAVETAA | 357.2 |
| Rv0280_19 | PQPDSAAAAAAAAAVRD | 464.8 | *Rv0293c_01* | GLSGFAEAMAQAVYDFR | 356.8 |
| Rv3478_01 | INSARMYAGPGSASLVA | 463.1 | *Rv1196_04* | GQAELTAAQVRVAAAAY | 354.6 |
| Rv2823c_02 | RLFIATGSVPLAANDLM | 462.8 | *Rv0280_05* | YQAVAEAAVASAPQTTP | 352.3 |
| Rv0291_03 | DTTPRNVAFAGAAALSV | 462.2 | *Rv3873_04* | RMWNQAALAMEVYQAET | 350.8 |
| Rv1808_06 | SAVVAQAAGAAASSDIT | 458.1 | *Rv1802_02* | AAPYVAWMSATAALARE | 349.1 |
| Rv1196_04 | GQAELTAAQVRVAAAAY | 454.6 | *Rv0280_04* | AYTTAVAAMPTLVELAA | 347.8 |
| Rv0280_06 | GLAGMQPAAIPALAPVA | 452.9 | *Rv2874_04* | GSAVFDYPPSLAANSFA | 345.8 |
| Rv3478_04 | YETAYRLTVPPPVIAEN | 452.7 | *Rv1808_06* | SAVVAQAAGAAASSDIT | 345.2 |
| Rv0280_07 | MQPAAIPALAPVAAAPS | 452.3 | *Rv1800_02* | AVSVFEEALAATVHPAM | 343.5 |
| Rv1706c_09 | YWMMFLGALATAEGFIY | 451.9 | *Rv0987_04* | TMFLFLASGANSGALID | 343.4 |
| Rv1802_08 | VAANRAELAVLAATNIF | 451.8 | *Rv1361c_02* | ASDLFSAASAFQSVVWG | 342.7 |
| Rv1172c_02 | NAANVVAAVPTTGVLAA | 449.4 | *Rv1317c_01* | RNVRFLPTAAAAQGEGF | 341.1 |
| Rv1317c_01 | RNVRFLPTAAAAQGEGF | 449.2 | *Rv1361c_03* | AGLMVAAASPYVAWMSV | 340.2 |
| Rv1172c_07 | AGAAAAAALPALAAESI | 448.4 | *Rv3876_04* | QPRSYLAPPTRPAPTEP | 335.5 |
| Rv1196_03 | AGLMVAAASPYVAWMSV | 447.2 | *Rv0124_05* | GAGAYAAAEAQVEQQLL | 335.3 |
| Rv0297_03 | GGAIFNAGTPGAAGTGG | 446.7 | *Rv3873_01* | LNTARLMAGAGPAPMLA | 333.9 |
| Rv2892c_02 | ELTGAYWAGPAAASMVA | 445.7 | *Rv2123_04* | VLDPLIYFGPFAPLTSP | 332.7 |
| Rv0987_02 | RSLSRTSIAIAALMMAV | 442.2 | *Rv1387_01* | EMAAQQHVVIEAYTAAV | 332.3 |
| Rv3125c_08 | AATAFEAALAATVHPTA | 441.9 | *Rv0124_03* | SAANAAAAAPTTAVLAA | 331.8 |
| Rv1706c_01 | PFVGWLSTTATLAEQAA | 440.1 | *Rv0442c_03* | ELTSGAWLGPSAAAMMA | 331.4 |
| Rv3125c_05 | TVASQAQLAAIQARAAA | 439.9 | *Rv3621c_06* | VPAVWTATTPAASPAVL | 331.3 |
| Rv0278c_04 | GLDGFNAPASTSPLHTL | 439.9 | *Rv0278c_02* | LGSSISAANAAAAANTT | 331.2 |
| Rv0286_04 | AYTTALAAMPTLAELAA | 436.9 | *Rv0280_08* | AAIPALAPVAAAPSTLP | 330.5 |
| Rv1387_09 | APAPAAAAGATAAGPTP | 435.8 | *Rv1243c_02* | AAGSYAAAEAANASPMQ | 329.5 |
| Rv0280_16 | AAVASAAAPASAPAAST | 435.4 | *Rv0289_01* | WVSTFSPGTPFAIAVAI | 325.8 |
| Rv0124_04 | HAQAYQALSAQAAAFHQ | 433.1 | *Rv1317c_03* | PLALARAQRMQTARVLI | 325.6 |
| Rv0124_01 | AGIGSAISAANAAAAAP | 430.8 | *Rv0256c_03* | AYGTALAAMPTLAELGA | 323.3 |
| Rv1789_01 | VNSVRMYAGPGSAPMVA | 430.6 | *Rv3020c_02* | SAAAFQGAHARFVAAAA | 322.3 |
| Rv1800_01 | SARVFAGAGSAPMLAAA | 428.0 | *Rv2875_01* | AATSFAAAGLAALAVAV | 322.1 |
| Rv0290_01 | DLLVLQPVPAGPAAPGI | 427.9 | *Rv1387_02* | AGLSGLSAIPSAAIPAV | 319.0 |
| Rv2123_03 | VAHESVAATPSTPPAPQ | 424.0 | *Rv0287_02* | SSAAFQAAHARFVAAAA | 318.5 |
| Rv0256c_07 | VSTAAVAAAPQTTPAPQ | 423.5 | *Rv0286_04* | AYTTALAAMPTLAELAA | 316.4 |
| Rv0292_01 | RTTVLVWVGPPASDTNV | 422.0 | *Rv1802_11* | QVTPFAAPPPTTNAAGL | 316.0 |
| Rv0442c_09 | SSAGYATGGMSTAALSS | 420.9 | *Rv0286_08* | YQAASGAALASAPRTVP | 309.8 |
| Rv2892c_12 | GPTIFAGPRASPSVAGG | 419.8 | *Rv0453_03* | VTAAYAAALVAMPTLAE | 307.0 |
| Rv0286_10 | LPIVLAPAVIPPASTPL | 418.7 | *Rv3136_02* | SARMYTGPGAGSLLAAA | 306.6 |
| Rv1705c_08 | SWRALGWIGPEAAEAAA | 418.0 | *Rv0280_15* | PGAAVASAAAPASAPAA | 306.2 |
| Rv2608_04 | KYPLNVFATANAIAGIL | 417.6 | *Rv1243c_01* | NAQAYQALSAQAAAFHQ | 305.8 |
| Rv0278c_01 | MSFVIAAPEVIAAAATD | 416.5 | *Rv0280_10* | STLPAVAMAPTMAAPGA | 303.8 |
| Rv0286_12 | APAPAAPATASFAYAVG | 416.2 | *Rv1366_01* | EYPGQRAMALGEASKNK | 302.8 |
| Rv1243c_01 | NAQAYQALSAQAAAFHQ | 416.2 | *Rv3125c_10* | QVGTQVAGMATTASAAV | 301.7 |
| Rv0287_01 | GQAEQAAMSAQAFHQGE | 413.1 | *Rv0278c_03* | GGGAYAAAEAAAVSPLL | 297.4 |
| Rv0442c_03 | ELTSGAWLGPSAAAMMA | 412.9 | *Rv1172c_05* | PRAGAAAAAALPALAAE | 297.2 |
| Rv0280_17 | GAAGFGYPYAIAPPGIG | 411.8 | *Rv2892c_08* | ELTPFTAAPVTTSPAAL | 297.2 |
| Rv1789_04 | AAAAFEAAFAATVPPPL | 410.8 | *Rv1705c_03* | YETAFAAIVPPPLIAAN | 295.2 |
| Rv0280_08 | AAIPALAPVAAAPSTLP | 410.1 | *Rv1706c_10* | SAWSQLGAGPVAASATL | 294.3 |
| Rv0297_01 | IRSAINAANAAAAAQTT | 409.9 | *Rv0286_10* | LPIVLAPAVIPPASTPL | 292.1 |
| Rv1387_02 | AGLSGLSAIPSAAIPAV | 409.7 | *Rv1789_04* | AAAAFEAAFAATVPPPL | 290.1 |
| Rv1172c_06 | RAGAAAAAALPALAAES | 407.9 | *Rv1705c_06* | TATKVTPFAPPPNTTSP | 288.6 |
| Rv1886c_01 | HPQQFIYAGSLSALLDP | 406.8 | *Rv3024c_03* | RAPVFTAGAAPSGPVDC | 286.7 |
| Rv0256c_05 | ARMWIQAATTMASYQAV | 403.9 | *Rv0286_06* | RMWLQAAAVMGLYQAAS | 284.9 |
| Rv0280_12 | PAVAMAPTMAAPGAAVA | 403.8 | *Rv0291_02* | YAAGYVSGVAALVRSRY | 283.1 |
| Rv0286_11 | PSAGAAPAPAAPATASF | 401.6 | *Rv0280_09* | PALAPVAAAPSTLPAVA | 282.8 |
| Rv3804c_03 | HPQQFVYAGAMSGLLDP | 401.5 | *Rv1387_07* | AAPGMLPASAPAPAAAA | 282.7 |
| Rv0256c_09 | PLAPLTLAPASAAGGFA | 399.2 | *Rv0279c_03* | GGGAYAAAEAAATSPLL | 280.5 |
| Rv0279c_02 | LESSIAAANAAAAANTT | 398.7 | *Rv0124_01* | AGIGSAISAANAAAAAP | 278.7 |
| Rv2123_05 | PLIYFGPFAPLTSPVLL | 396.0 | *Rv0292_02* | LLTRLHWPTPTAGAHRA | 278.2 |
| Rv1706c_08 | VLTPFAPPPQTTNATGL | 392.5 | *Rv0256c_02* | AATAYGTALAAMPTLAE | 278.0 |
| Rv0256c_10 | GLAGLVGIPPSAPPVIP | 390.8 | *Rv0124_02* | IGSAISAANAAAAAPTT | 278.0 |
| Rv3621c_05 | ALPAAVPAIPSAGLSGV | 389.6 | *Rv1196_11* | LRVPPRPYVMPHSPAAG | 277.7 |
| Rv0453_05 | ATPTTAPASAPAAGAAP | 385.7 | *Rv0453_05* | ATPTTAPASAPAAGAAP | 275.4 |
| Rv1789_06 | SAAMYAYAGSSASASAV | 385.5 | *Rv3018c_01* | TAPVWLASPPEVHSALL | 273.6 |
| Rv1802_07 | YEAAFAATVPPPVVAAN | 385.1 | *Rv0453_04* | NPQLLVAALTPAISGLG | 270.4 |
| Rv2823c_01 | MFNRFGSGTANLAFAPE | 384.5 | *Rv0278c_01* | MSFVIAAPEVIAAAATD | 270.2 |
| Rv0987_04 | TMFLFLASGANSGALID | 384.2 | *Rv0453_01* | SALIWMASPPEVHSALL | 269.8 |
| Rv0256c_06 | YQAVSTAAVAAAPQTTP | 382.9 | *Rv3136_06* | AAAMYGYATASAAAALL | 269.6 |
| Rv1802_12 | FQKFFNPVTPFNPDLIP | 381.0 | *Rv1789_01* | VNSVRMYAGPGSAPMVA | 267.8 |
| Rv0292_02 | LLTRLHWPTPTAGAHRA | 380.7 | *Rv0129c_02* | QQFPYAASLSGFLNPSE | 265.5 |
| Rv1196_05 | YETAYGLTVPPPVIAEN | 380.3 | *Rv0279c_01* | MSFVIAAPEVIAAAATD | 264.8 |
| Rv1387_04 | APPMLAVAGVGPAVAAP | 380.0 | *Rv3125c_11* | TQVAGMATTASAAVTPV | 260.9 |
| Rv0442c_10 | LTNTPVAAPASAPVGGL | 379.5 | *Rv0290_02* | LVLQPVPAGPAAPGIVE | 259.2 |
| Rv0290_03 | VVAFFTAAAVVGASVAL | 376.7 | *Rv0287_01* | GQAEQAAMSAQAFHQGE | 256.4 |
| Rv1387_10 | AGTVRKEAVVKAAGLTT | 376.2 | *Rv2892c_06* | AAAMYAYAGSAAIATEL | 255.7 |
| Rv1243c_02 | AAGSYAAAEAANASPMQ | 374.8 | *Rv3125c_09* | TLAPFSLPPVSLAGLAA | 253.7 |
| Rv3876_02 | PSASFPPAPASANLPKP | 374.6 | *Rv1196_07* | AAAMFGYAAATATATAT | 253.3 |
| Rv1808_04 | AMYGYAGSSATASQLAP | 372.2 | *Rv2123_06* | DALGFAGTIPKSAPGSA | 253.3 |
| Rv1196_09 | LSSMLKGFAPAAAAQAV | 372.2 | *Rv0291_03* | DTTPRNVAFAGAAALSV | 252.4 |
| Rv1705c_09 | RALGWIGPEAAEAAAAA | 370.7 | *Rv1361c_08* | AMFGYAATAATATEALL | 246.7 |
| Rv2892c_07 | AMYAYAGSAAIATELTP | 366.3 | *Rv1706c_02* | TLAEQAAMQARAAAAAF | 245.2 |
| Rv1196_02 | ASDLFSAASAFQSVVWG | 363.4 | *Rv1789_02* | VAPYVAWMSAAAAQAEQ | 243.2 |
| Rv0278c_02 | LGSSISAANAAAAANTT | 361.5 | *Rv1789_11* | GLGGMLGGGPVAAGLGN | 243.0 |
| Rv2123_01 | YWLTHAATVATAAAAAH | 361.3 | *Rv1705c_04* | AMAMYGYAGSSATATKV | 242.9 |
| Rv1361c_06 | TAYGLTVPPPVIAENRA | 358.7 | *Rv1705c_10* | EAAEAAAAAPAAVGAAV | 237.6 |
| Rv1196_11 | LRVPPRPYVMPHSPAAG | 358.5 | *Rv1802_06* | AAAAYEAAFAATVPPPV | 236.6 |
| Rv1802_11 | QVTPFAAPPPTTNAAGL | 357.9 | *Rv1808_01* | AAGVYETAFAATVPPPV | 234.9 |
| Rv0442c_02 | SALMFAGPGSGPLIAAA | 355.5 | *Rv1802_03* | PYVAWMSATAALAREAA | 234.5 |
| Rv1706c_10 | SAWSQLGAGPVAASATL | 354.8 | *Rv0987_03* | KSDVYVSPPTLTSGRPS | 234.3 |
| Rv3021c/ Rv3022c_02 | LVSAVEPAPASTSVSVL | 353.0 | *Rv0286_07* | VMGLYQAASGAALASAP | 231.6 |
| Rv0290_04 | VGWYLVAATAAAATLRA | 350.7 | *Rv2123_01* | YWLTHAATVATAAAAAH | 230.3 |
| Rv0286_14 | APAATVPAAAAAAATRG | 347.9 | *Rv1387_08* | MLPASAPAPAAAAGATA | 229.6 |
| Rv0280_10 | STLPAVAMAPTMAAPGA | 346.5 | *Rv3135_04* | AAAAFEQAHAMTVPPAL | 228.8 |
| Rv1317c_03 | PLALARAQRMQTARVLI | 346.0 | *Rv0280_14* | APGAAVASAAAPASAPA | 228.2 |
| Rv0280_02 | QTGAWQGPSAAAYVAAH | 341.7 | *Rv0280_20* | QPDSAAAAAAAAAVRDQ | 226.0 |
| Rv2874_01 | VESRRAAAAASAYASRC | 339.6 | *Rv0297_01* | IRSAINAANAAAAAQTT | 225.9 |
| Rv1172c_01 | MSFVFAAPEALAAAAAD | 338.5 | *Rv1361c_11* | LRMPPRAYVMPRVPAAG | 225.2 |
| Rv0278c_03 | GGGAYAAAEAAAVSPLL | 335.3 | *Rv1788_01* | HAQIYQAVSAQAAAIHE | 225.1 |
| Rv0280_18 | FGYPYAIAPPGIGFGSG | 331.8 | *Rv1800_03* | MAGYYVGASAVATQLAS | 224.7 |
| Rv1802_03 | PYVAWMSATAALAREAA | 330.0 | *Rv0278c_04* | GLDGFNAPASTSPLHTL | 219.8 |
| Rv0280_13 | MAPTMAAPGAAVASAAA | 329.6 | *Rv0280_18* | FGYPYAIAPPGIGFGSG | 219.8 |
| Rv3478_02 | ASDLFSAASAFQSVVWG | 328.3 | *Rv1802_04* | TAALAREAAAQASAAAA | 219.4 |
| Rv1705c_11 | LPPNWAGASPSLAPTVG | 324.9 | *Rv1387_10* | AGTVRKEAVVKAAGLTT | 218.9 |
| Rv0256c_11 | PVAAIAPSIPTPTPTPA | 323.6 | *Rv1800_04* | PAYGYSTSPPNVATPFG | 218.5 |
| Rv2853_01 | FHSRFVQALTTAAASYA | 321.1 | *Rv1886c_01* | HPQQFIYAGSLSALLDP | 217.9 |
| Rv0124_02 | IGSAISAANAAAAAPTT | 320.9 | *Rv0129c_01* | IKVQFQGGGPHAVYLLD | 217.8 |
| Rv1361c_09 | LHSMLKGFAPAAAQAVE | 318.0 | *Rv1706c_11* | LGAGPVAASATLAAKIG | 214.6 |
| Rv1705c_10 | EAAEAAAAAPAAVGAAV | 317.5 | *Rv1802_10* | AMYGYAGSSSVATQVTP | 210.6 |
| Rv0442c_07 | IATAFEAALAATVQPAV | 316.8 | *Rv1317c_05* | AAARFESATASAGTVSL | 206.0 |
| Rv0289_01 | WVSTFSPGTPFAIAVAI | 315.4 | *Rv2874_01* | VESRRAAAAASAYASRC | 203.5 |
| Rv1808_02 | YETAFAATVPPPVIEAN | 313.6 | *Rv1361c_07* | AAAMFGYAATAATATEA | 201.7 |
| Rv3136_02 | SARMYTGPGAGSLLAAA | 313.6 | *Rv0290_03* | VVAFFTAAAVVGASVAL | 201.4 |
| Rv2892c_09 | TVSSTVPPLATTAAVPQ | 311.5 | *Rv1705c_01* | YVAWMRATAIQAEQAAS | 201.1 |
| Rv2123_02 | THAATVATAAAAAHETA | 309.5 | *Rv1705c_05* | AMYGYAGSSATATKVTP | 197.6 |
| Rv1789_11 | GLGGMLGGGPVAAGLGN | 309.4 | *Rv0256c_04* | NESDYARMWIQAATTMA | 197.2 |
| Rv1808_05 | ATAAQSAVVAQAAGAAA | 309.1 | *Rv2892c_11* | SWGPTIFAGPRASPSVA | 196.3 |
| Rv0279c_04 | GRDGFNAPASTPLHTLQ | 308.0 | *Rv2892c_09* | TVSSTVPPLATTAAVPQ | 194.7 |
| Rv3804c_02 | YSDWYQPACGKAGCQTY | 307.0 | *Rv1172c_06* | RAGAAAAAALPALAAES | 194.1 |
| Rv3876_04 | QPRSYLAPPTRPAPTEP | 304.5 | *Rv1387_06* | VGPAVAAPGMLPASAPA | 189.7 |
| Rv3804c_01 | IKVQFQSGGANSPALYL | 304.2 | *Rv0297_02* | HAQAYQAASAQAAAFHA | 188.2 |
| Rv1361c_10 | MLKGFAPAAAQAVETAA | 300.0 | *Rv1705c_09* | RALGWIGPEAAEAAAAA | 186.6 |
| Rv0280_15 | PGAAVASAAAPASAPAA | 299.6 | *Rv2892c_12* | GPTIFAGPRASPSVAGG | 181.8 |
| Rv1802_05 | REAAAQASAAAAAYEAA | 294.7 | *Rv1706c_04* | FEAAFAMTVPPPAIAAN | 179.8 |
| Rv0124_03 | SAANAAAAAPTTAVLAA | 294.5 | *Rv1791_01* | HAQMYQTVSAQAAAIHE | 173.5 |
| Rv1789_05 | FEAAFAATVPPPLIAAN | 293.4 | *Rv0290_04* | VGWYLVAATAAAATLRA | 172.9 |
| Rv2892c_06 | AAAMYAYAGSAAIATEL | 291.0 | *Rv1789_06* | SAAMYAYAGSSASASAV | 171.2 |
| Rv1317c_04 | LARAQRMQTARVLIETT | 290.6 | *Rv0280_19* | PQPDSAAAAAAAAAVRD | 168.3 |
| Rv0124_05 | GAGAYAAAEAQVEQQLL | 289.8 | *Rv1387_09* | APAPAAAAGATAAGPTP | 168.3 |
| Rv0442c_08 | AGYHFDASAAVAQLAPW | 289.7 | *Rv1441c_01* | SVGSYAAAEAANASPLQ | 167.5 |
| Rv3621c_06 | VPAVWTATTPAASPAVL | 288.8 | *Rv2874_02* | TSQRSLATPPTISVPSG | 164.3 |
| Rv1706c_11 | LGAGPVAASATLAAKIG | 286.2 | *Rv3136_01* | VNSARMYTGPGAGSLLA | 164.0 |
| TB10.4 | ASDLFSAASAFQSVVWG | 278.9 | *Rv3125c_04* | LSTVASQAQLAAIQARA | 157.9 |
| Rv1361c_08 | AMFGYAATAATATEALL | 278.7 | *Rv0286_14* | APAATVPAAAAAAATRG | 157.3 |
| Rv1789_09 | WLWQILFGTPNFPTSIS | 274.4 | *Rv0124_04* | HAQAYQALSAQAAAFHQ | 154.4 |
| Rv1361c_07 | AAAMFGYAATAATATEA | 262.9 | *Rv1706c_05* | AFAMTVPPPAIAANRTL | 149.8 |
| Rv1317c_02 | LLQAVVGAGPLALARAQ | 261.9 | *Rv1361c_06* | TAYGLTVPPPVIAENRA | 149.6 |
| Rv0256c_08 | APQIVKANAPTAASDEP | 261.0 | *Rv1361c_09* | LHSMLKGFAPAAAQAVE | 140.6 |
| Rv1808_01 | AAGVYETAFAATVPPPV | 256.9 | *Rv1706c_01* | PFVGWLSTTATLAEQAA | 135.3 |
| None | INSARMYAGPGSASLVA | 251.2 | *Rv1789_05* | FEAAFAATVPPPLIAAN | 135.2 |
| Rv3876_03 | LSERFVSAPPPPPPPPP | 247.1 | *Rv1172c_04* | ALLPRAGAAAAAALPAL | 131.2 |
| Rv2874_02 | TSQRSLATPPTISVPSG | 234.0 | *Rv1706c_09* | YWMMFLGALATAEGFIY | 130.5 |
| Rv3136_01 | VNSARMYTGPGAGSLLA | 231.6 | *Rv1789_07* | AMYAYAGSSASASAVTP | 129.1 |
| Rv0291_04 | RNVAFAGAAALSVLVGL | 227.7 | *Rv1706c_08* | VLTPFAPPPQTTNATGL | 126.0 |
| Rv0987_03 | KSDVYVSPPTLTSGRPS | 222.9 | *Rv1789_10* | KTLGLIGSAAPAAVAAA | 126.0 |
| Rv2123_06 | DALGFAGTIPKSAPGSA | 216.5 | *Rv1789_12* | LSVPPVWSGPLPGSVTP | 117.3 |
| Rv1788_01 | HAQIYQAVSAQAAAIHE | 215.5 | *Rv1705c_11* | LPPNWAGASPSLAPTVG | 111.2 |
| Rv1361c_11 | LRMPPRAYVMPRVPAAG | 186.5 | *Rv1705c_08* | SWRALGWIGPEAAEAAA | 108.3 |
| Rv1802_06 | AAAAYEAAFAATVPPPV | 178.7 | *Rv1705c_07* | KVTPFAPPPNTTSPSAA | 105.6 |
| Rv1789_10 | KTLGLIGSAAPAAVAAA | 175.7 | *Rv1196_09* | LSSMLKGFAPAAAAQAV | 101.1 |
| Rv3876_05 | VINHIMPGEPNVAVKDL | 168.9 | *Rv1172c_03* | SADAYATAEASAAQTMV | 99.4 |
| Rv1802_04 | TAALAREAAAQASAAAA | 167.5 | *Rv0290_01* | DLLVLQPVPAGPAAPGI | 98.7 |
| Rv2874_04 | GSAVFDYPPSLAANSFA | 154.7 | *Rv1789_08* | AQGTQAAAVATAAGTAQ | 73.8 |
| Rv2874_03 | ILAAIVVAGATATIGLG | 139.5 | *Rv1706c_07* | AMYGYASAAAPATVLTP | 73.6 |
| Rv1789_08 | AQGTQAAAVATAAGTAQ | 137.4 | *Rv1705c_02* | AAAAYETAFAAIVPPPL | 67.8 |
| Rv2875_01 | AATSFAAAGLAALAVAV | 112.1 | *Rv1789_09* | WLWQILFGTPNFPTSIS | 53.2 |
