# Supplementary figures and images for: Rescuing ESAT-6 Specific CD4 T Cells From Terminal Differentiation Is Critical for Long-Term Control of Murine Mtb Infection
Source: Front Immunol. 2020 Nov 6;11:585359. doi: 10.3389/fimmu.2020.585359 (PMC7677256; doi:10.3389/fimmu.2020.585359)

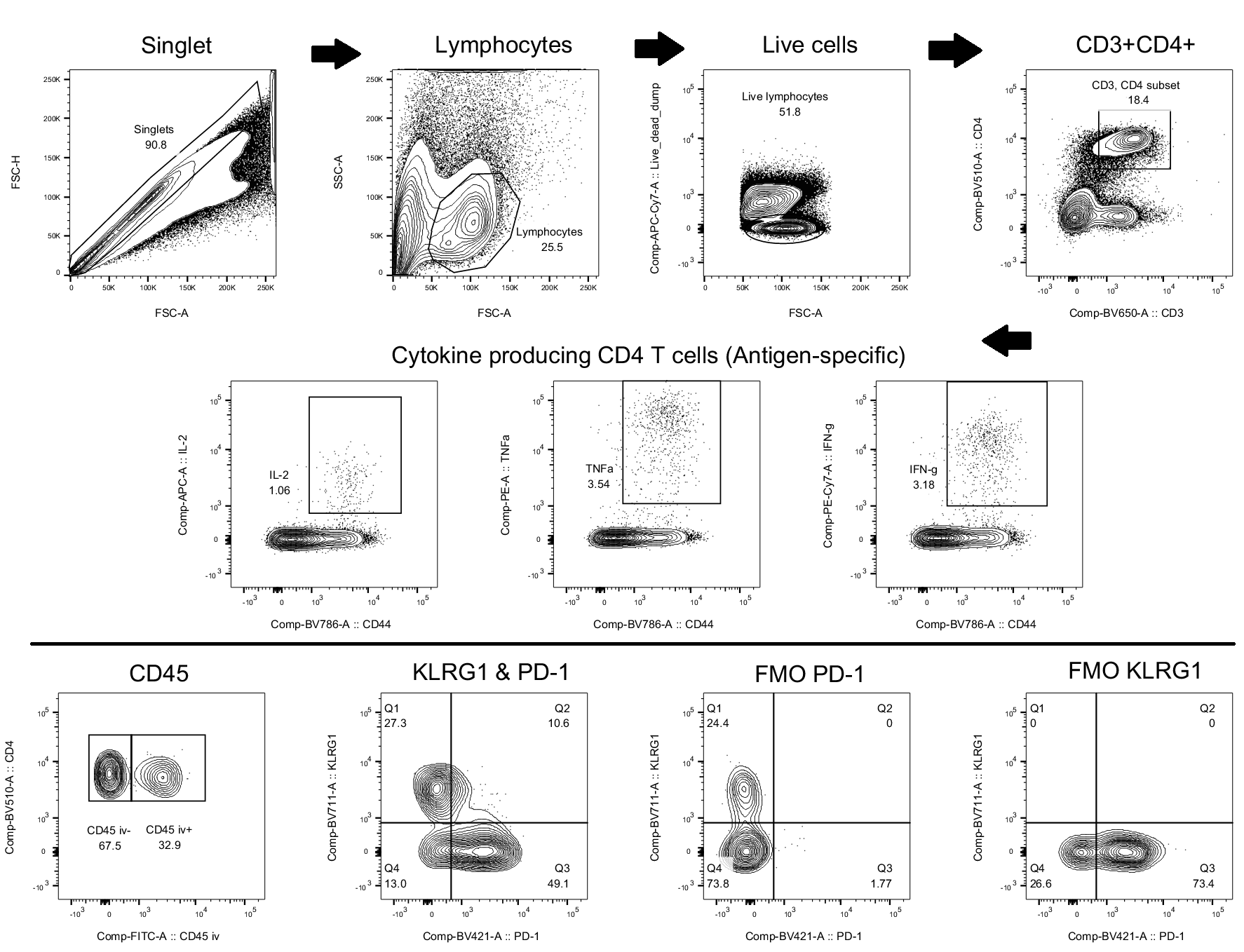

Supplement: Supplementary Figure 1 — Gating strategy for antigen-specific CD4 T cells. Single-cell suspensions were prepared from the spleens or lungs of vaccinated or infected mice. Single cells were initially gated from a FSC-A and FSC-H plot. Lymphocytes were afterwards gated from a SSC-A and FSC-A plot. Non-viable cells were excluded by staining with fixable eflour-780 dye. CD3 and CD4 double positive T cells were gated followed by a gate for CD44high and TNFα, IL-2 and IFN-γ positive CD4 T cells. Boolean gating was performed on all samples, combining CD4 T cells producing either TNFα, IL-2 or IFN-γ into one group (antigen-specific CD4 T cells). Antigen-specific CD4 T cells were further characterized for their expression of KLRG1 and PD-1 and CD45. Only the KLRG1+PD-1- subset is visualized in the corresponding figures. Combination gates for TNFα, IL-2, or IFN-γ producing CD4 T cells were created and the T cell differentiation state expressed as the functional differentiation score (FDS). FDS represents the ratio of all IFN-γ producing CD4 T cell subsets divided by subsets producing other cytokines (IL-2, TNFα), but not IFN-γ (high FDS = high IFN-γ production). Fluorescence minus one controls for KLRG1 and PD-1 are shown. [file Image_1.tiff]

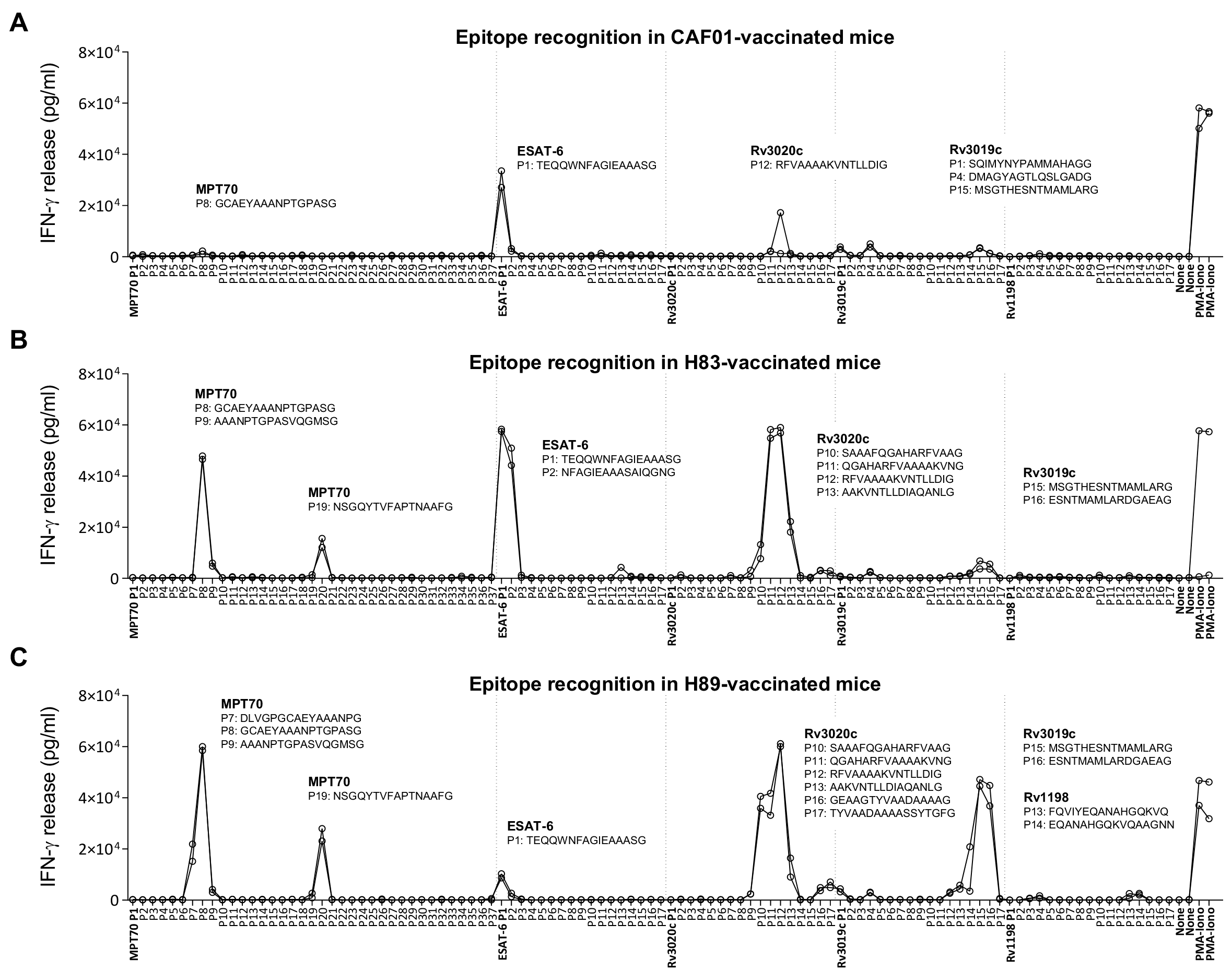

Supplement: Supplementary Figure 2 — Epitope recognition pattern in H83 and H89 vaccinated mice. CB6F1 mice were immunized three times with CAF01 adjuvant alone, 0.5 µg recombinant H83 or H89. Six weeks after third immunization, mice were challenged with Mtb Erdman. Four weeks post Mtb infection, lung cells from two mice were harvested and pooled within each group. Technical duplicates of pooled lungs cells were in vitro restimulated using 16-mer peptides with 11 amino acid overlap spanning all five antigens comprising H83 and H89. As control, lung cells were stimulated with media only (None) or phorbol 12-myristate 13-acetate and ionomycin (PMA-Iono). Culture supernatants were harvested after 3 days of culture and analyzed for levels of IFN-γ by ELISA. Epitopes with the highest responses are indicated with the peptide sequence used for stimulation. [file Image_2.tiff]

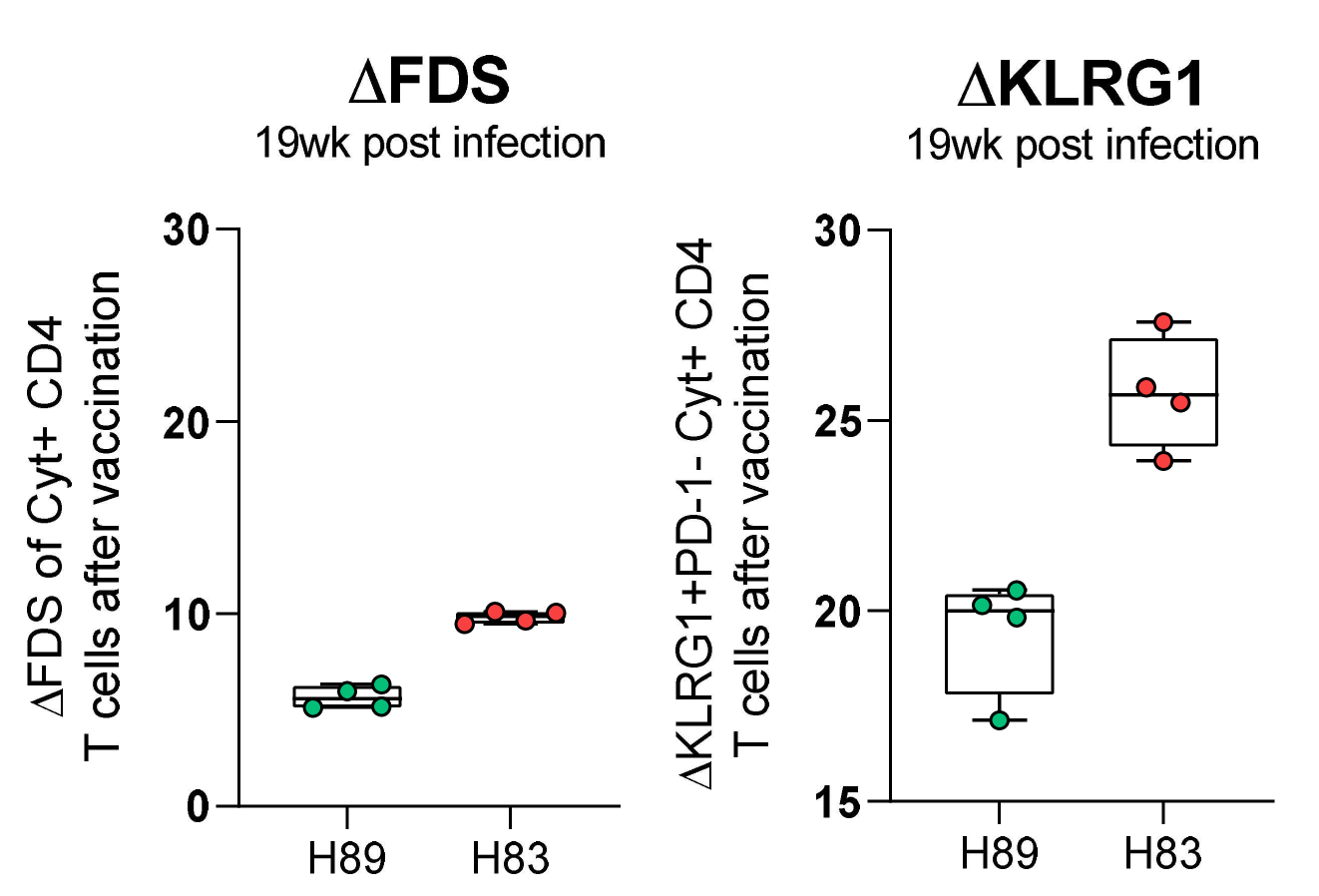

Supplement: Supplementary Figure 3 — H83-vaccinated mice display a greater reduction in FDS and KLRG1-expressing CD4 T cells after vaccination. The difference in functional differentiation score (FDS) (left) and KLRG1 expression (right) of H83- and H89-specific CD4 T cells in respectively H83- and H89-vaccinated mice compared to saline mice (n = 4). FDS represents the ratio of all IFN-γ producing CD4 T cell subsets divided by subsets producing other cytokines (IL-2, TNFα), but not IFN-γ (high FDS = high IFN-γ production). Delta FDS values and KLRG1+PD-1- were calculated as the mean value for H83 and H89-specific CD4 T cells in infected mice subtracted the value for H83-specific CD4 T cells in H83 vaccinated mice and H89-specific CD4 T cells in H89 vaccinated mice 19 week post infection. Graphs are visualized as box plots showing all points with whiskers indicating minimum and maximum values. [file Image_3.tiff]
